# Pyrroline‐5‐carboxylate reductase 1 interacts with Nudix hydrolase 5 to drive bladder cancer malignant progression via activation of the APOE/JAK2/STAT3 signalling axis

**DOI:** 10.1002/ctm2.70840

**Published:** 2026-09-18

**Authors:** Haotian Chen, Shuya Ji, Pengfei Wu, Ji Liu, Wentao Zhang, Yadong Guo, Jingcheng Zhang, Zhijing Zhang, YanLing Liu, Yongqiang Liu, Chengqi Jin, Sichen Di, Yang Wu, Junfeng Zhang, Xiaojun Zhu, Shiyu Mao, Bing Shen, Xudong Yao

**Affiliations:** ^1^ Department of Urology Shanghai Tenth People's Hospital Tongji University School of Medicine Shanghai China; ^2^ Urologic Cancer Institute Tongji University School of Medicine Shanghai China; ^3^ School of Medicine Tongji University Shanghai China; ^4^ Department of Oncology Gongli Hospital of Shanghai Pudong New Area Shanghai China; ^5^ State Key Laboratory of Pollution Control and Resources Reuse Tongji Advanced Membrane Technology Centre Tongji University Shanghai China; ^6^ Department of Urology The Affiliated Hospital of Inner Mongolia Medical University Hohhot China

**Keywords:** APOE, bladder cancer, NUDT5, PYCR1, TH5427

## Abstract

**Background:**

Bladder cancer (BCa) is one of the most common urological malignancies, with a steadily increasing global incidence. Pyrroline‐5‐carboxylate reductase 1 (PYCR1) is a metabolic regulator with noncanonical oncogenic functions; however, its protein interaction network and downstream mechanisms in BCa remain incompletely understood.

**Methods:**

PYCR1 expression and its clinical significance were evaluated using the Cancer Genome Atlas (TCGA) and Gene Expression Omnibus (GEO) datasets and tissue microarrays. Cell counting kit‐8, colony formation, EdU, 3D Transwell experiments and xenograft models were used to investigate the biological functions of PYCR1. RNA sequencing, Western blot and quantitative polymerase chain reaction (qPCR) were performed to identify its downstream targets. Mass spectrometry combined with co‐immunoprecipitation identified Nudix hydrolase 5 (NUDT5) as a PYCR1‐interacting protein. Nuclear–cytoplasmic fractionation and adenosine triphosphate (ATP) quantification were used to measure nuclear ATP levels.

**Results:**

PYCR1 was upregulated in BCa and promoted tumour proliferation and migration in vitro and in vivo. Mechanistically, PYCR1 interacted with NUDT5 and promoted NUDT5‐dependent nuclear ATP generation. Wild‐type NUDT5 restored Apolipoprotein E (APOE) expression in PYCR1‐knockdown cells, whereas a catalytically inactive NUDT5 mutant failed to do so. RNA sequencing, gene set enrichment analysis, Western blot, and qPCR demonstrated that PYCR1 activated the APOE/JAK2/STAT3 signalling axis. Transcription factor prediction and dual‐luciferase reporter assays identified SP1 as a transcriptional regulator of APOE. Moreover, pharmacological inhibition of NUDT5 with TH5427 suppressed BCa progression and JAK2/STAT3 activation in vitro and inhibited xenograft growth in vivo.

**Conclusions:**

PYCR1 interacts with NUDT5 to increase nuclear ATP levels, thereby sustaining SP1‐dependent APOE transcription and activating JAK2/STAT3 signalling, ultimately promoting BCa progression. TH5427 may represent a promising therapeutic candidate for BCa.

## Key points

1. PYCR1, via NUDT5‐dependent nuclear ATP production, promotes SP1‐mediated APOE transcription.
2. The APOE activates the JAK2/STAT3 signaling pathway, thereby promoting proliferative and metastatic capacities of BCa cells.
3. TH5427, a small‐molecule inhibitor targeting NUDT5 enzymatic activity, effectively counteracts this process.

## Graphical abstract

Pyrroline‐5‐carboxylate reductase 1, via Nudix hydrolase 5 (NUDT5)‑dependent nuclear ATP production, promotes SP1‑mediated Apolipoprotein E (APOE) transcription. APOE, in turn, activates the JAK2/STAT3 signalling pathway, thereby promoting proliferative and metastatic capacities of bladder cancer cells. TH5427, a small‐molecule inhibitor targeting NUDT5 enzymatic activity, effectively counteracts this process.

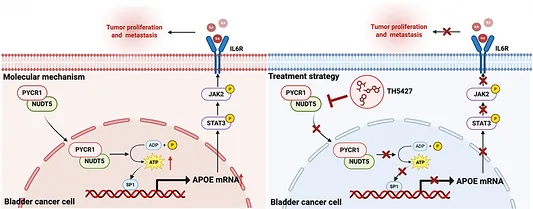

## 1 INTRODUCTION

Bladder cancer (BCa) ranks among the most prevalent malignant neoplasms within the urinary system. Clinically, 75% of patients present with non‐muscle‐invasive bladder cancer (NMIBC), while the remaining 25% are diagnosed with muscle‐invasive bladder cancer (MIBC).^1^ At present, platinum‐based chemotherapy remains the mainstay therapeutic regimen for advanced BCa, with the 5‐year survival rate typically ranging from 5% to 20%.^2^ Tumour recurrence and metastasis constitute the primary causes of mortality among BCa patients, yet the molecular mechanisms underlying the initiation and progression of this malignancy remain incompletely elucidated.

Tumour metabolic reprogramming is a key factor contributing to tumour recurrence, drug resistance, and metastasis.^3^ As a pivotal cofactor for proteins governing diverse physiological processes, proline metabolic dysregulation has been increasingly implicated in tumorigenesis and malignant progression by emerging studies.^4^, ^5^, ^6^ Pyrroline‐5‐carboxylate reductase 1 (PYCR1) serves as a rate‐limiting enzyme in proline biosynthesis, mediating the NADP‐dependent reduction of pyrroline‐5‐carboxylate to proline.^7^, ^8^ Accumulating evidence has demonstrated that PYCR1 is markedly overexpressed in an array of malignancies including hepatocellular carcinoma, breast cancer, lung cancer and prostate cancer, and its aberrant upregulation is closely correlated with tumour progression, metastasis and chemotherapeutic susceptibility.^9^, ^10^, ^11^, ^12^ In BCa, previous studies have demonstrated that PYCR1 promotes tumour progression through multiple mechanisms, including activation of the Akt/Wnt/β‐catenin pathway, interaction with EGFR to modulate EGFR/PI3K/AKT signalling and aerobic glycolysis, and regulation of PI3K/AKT signalling via BHLHE41‐mediated PYCR1 stability.^13^, ^14^, ^15^ A recent study further linked PYCR1 to SLC6A14/glutamine metabolism, glycolysis, and histone regulation.^16^ Although the metabolic functions and non‐canonical pro‐tumorigenic roles of PYCR1 have garnered increasing attention, the protein–protein interaction network underlying its non‐canonical functions in BCa remains incompletely understood. This study elucidates the potential molecular mechanisms underlying PYCR1‐mediated BCa progression and, for the first time, identifies Nudix hydrolase 5 (NUDT5) as a critical interacting protein of PYCR1. NUDT5 is a member of the NUDIX hydrolase family.^17^ Functioning as a pyrophosphorylase, it mediates the synthesis of nuclear adenosine triphosphate (ATP) from adenosine diphosphate (ADP)‐ribose in a pyrophosphate‐dependent manner.^18^ Nuclear ATP is essential for chromatin remodelling, DNA damage repair, transcriptional regulation and cellular proliferation.^19^ NUDT5 has been implicated in the malignant progression and therapeutic resistance of multiple neoplasms, most prominently breast cancer.^20^, ^21^, ^22^ Prior investigations have revealed that TH5427, a specific small‐molecule inhibitor targeting NUDT5, abolishes nuclear ATP production, thereby repressing chromatin remodelling, transcriptional regulation and the proliferation of breast cancer cells.^23^ Nevertheless, the biological significance of NUDT5 in BCa and the inhibitory efficacy of its targeted inhibitors against BCa progression remain largely unexplored.

Apolipoprotein E (APOE) is a pivotal plasma lipoprotein central to cholesterol transport and metabolic homeostasis.^24^ Although the regulatory roles of APOE in cholesterol balance, atherosclerosis, and Alzheimer's disease have been well characterised, its functional role in malignancies remains markedly underinvestigated.^25^ Recent studies have revealed that elevated APOE expression enhances glycolytic metabolism in thyroid carcinoma via modulating the IL‑6/JAK2/STAT3 signalling axis, thereby promoting malignant progression.^26^ In addition, a multicentre cohort study has identified urinary APOE as a candidate predictive biomarker for BCa.^27^ Notably, the precise biological functions and underlying molecular mechanisms of APOE in BCa remain to be fully elucidated.

This study demonstrates that PYCR1 interacts with NUDT5 and facilitates nuclear ATP generation. Elevated nuclear ATP levels activate SP1‐dependent APOE transcription and enhance JAK2/STAT3 signalling, ultimately promoting BCa progression. TH5427, a specific small‐molecule inhibitor targeting NUDT5, exerts antitumour efficacy in vitro and in vivo. Collectively, these findings uncover the non‐canonical biological role of PYCR1 in BCa and establish an innovative therapeutic approach.

## 2 MATERIALS AND METHODS

### 2.1 Data acquisition

Three independent datasets from our institution and public databases were included, including a BCa dataset from Shanghai Tenth People's Hospital (STPH), the BCa dataset from the Cancer Genome Atlas (TCGA; http://cancergenome.nih.gov/), and the GSE135077 BCa dataset from the Gene Expression Omnibus (GEO; https://www.ncbi.nlm.nih.gov/geo/). Using “metabolism” as the search keyword, the BIOSYNTHETIC_PROCESS gene set (including 470 genes) from the MSigDB database serves as a resource for metabolism‐associated genes. Patients enrolled in this study satisfied the following inclusion criteria:^1^ histologically confirmed diagnosis of BCa^2^; complete available clinical and prognostic data^3^; assessable RNA expression profiles. A total of 146 BCa patients were consecutively enrolled in the STPH dataset between October 2019 and July 2021. All procedures involving human participants were reviewed and approved by the Ethics Committee of STPH (No. 2021KN108). The RNA‐sequencing protocol employed at STPH was consistent with previously published reports.^28^, ^29^

### 2.2 3D Transwell invasion assays

This experiment was performed as described previously with minor modifications.^30^ Briefly, 1 × 10^5^ GFP‐expressing cancer cells were resuspended in 10 µL of 100% Matrigel, plated into 24‐well plates, and fluorescence images of the cell droplets were captured on days 0 and 6 using a fluorescence microscope. The culture medium was refreshed every 3 days. Cell migration out of the droplets was quantified via RFP signals using ImageJ software, while the GFP fluorescence signals of the cells were represented by an orange pseudocolour.

### 2.3 ATP concentration assays

Using BCa cells, the experimental group received TH5427 treatment, while the control group was supplemented with dimethyl sulfoxide (DMSO). ATP concentration was measured using an ATP assay kit (Beyotime) according to the manufacturer's instructions.^31^

### 2.4 Patient‐derived tumour organoids

Tumour specimens procured via transurethral resection or radical cystectomy were mechanically minced into 1–2 mm^3^ fragments, followed by enzymatic dissociation with collagenase type IV (40508ES60; Yeasen) in the presence of 10 µM Y‐27632 (HY‐10071; MCE) for 1 h. The resultant suspension was strained through a 70‐µm filter, centrifuged, and the pelleted cells were seeded into pre‐warmed 24‐well plates. After a 30‐min incubation at 37°C to facilitate organoid initiation, the Matrigel domes were solidified, and the cultures were subsequently maintained in human BCa organoid medium, which was renewed every 2–3 days. Organoid growth was monitored by tracking changes in spheroid diameter under bright‐field microscopy.

### 2.5 Nuclear–cytoplasmic fractionation

BCa cells in the logarithmic growth phase were collected, with approximately 5 × 10^6^–1 × 10^7^ cells used for each experiment. Cells were centrifuged at 500 × *g* for 3 min at 4°C, washed twice with ice‐cold phosphate‐buffered saline (PBS), and subjected to nuclear–cytoplasmic fractionation using a commercial kit (P0027; Beyotime, China). Briefly, the cell pellet was resuspended in 400 µl of ice‐cold cytoplasmic extraction buffer A and incubated on ice for 20–30 min with intermittent mixing. The lysate was then centrifuged at 1200 × *g* for 5 min at 4°C. The supernatant was collected as the cytoplasmic fraction, whereas the nuclear pellet was washed once with PBS and centrifuged at 2000 × *g* for 5 min at 4°C. The purified nuclear pellet was subsequently resuspended in 200 µl of nuclear storage buffer B. The obtained cytoplasmic and nuclear fractions were used for subsequent analyses.

### 2.6 Oligomycin treatment

BCa cells were seeded in six‐well plates and cultured to approximately 70%–80% confluence. Based on a previously reported ATP‐depletion protocol,^18^ cells were treated with oligomycin at a final concentration of 10 µM for 30 min, while an equal volume of DMSO was used as the vehicle control. Following treatment, the cells were rapidly washed twice with ice‐cold PBS and harvested for nuclear–cytoplasmic fractionation.

### 2.7 Gene set enrichment analysis

Using TCGA transcriptomic data, patients were stratified into PYCR1‐high and PYCR1‐low expression groups based on the median PYCR1 expression level. Gene set enrichment analysis (GSEA) was performed using the desktop version of GSEA software (version 4.2.3). Kyoto Encyclopaedia of Genes and Genomes (KEGG) and HALLMARK gene‐set collections were used to evaluate pathway enrichment between the PYCR1‐high and PYCR1‐low groups. For the si‐PYCR1 RNA‐sequencing dataset, GSEA was similarly performed between PYCR1‐knockdown and control cells. Pathways enriched in both the TCGA‐BLCA dataset and the si‐PYCR1 RNA‐sequencing dataset were subsequently compared to identify signalling pathways potentially regulated by PYCR1.

### 2.8 Statistical analysis

Statistical evaluations were carried out using R (v4.2.2) and GraphPad Prism (v9.1.1). Between‐group comparisons relied on two‐tailed Student's t‐tests for parametric data, whereas non‐parametric datasets were assessed via Mann‐Whitney U tests, following normality verification. Survival analyses of TCGA and GSE13507 datasets were performed using the BEST platform (https://rookieutopia.hiplot.com.cn/app_direct/BEST/). Patients were stratified according to the optimal cutoff value determined by Kaplan–Meier analysis and the log‐rank test. For the survival analysis of the STPH cohort, the optimal cutoff value was determined using the surv_cutpoint function in the “survminer” package in R, which is based on maximally selected rank statistics. Statistical significance was defined as *p* < 0.05.

## 3 RESULTS

### 3.1 PYCR1 is highly expressed in BCa tissues and correlates with unfavourable prognosis

**FIGURE 1:**
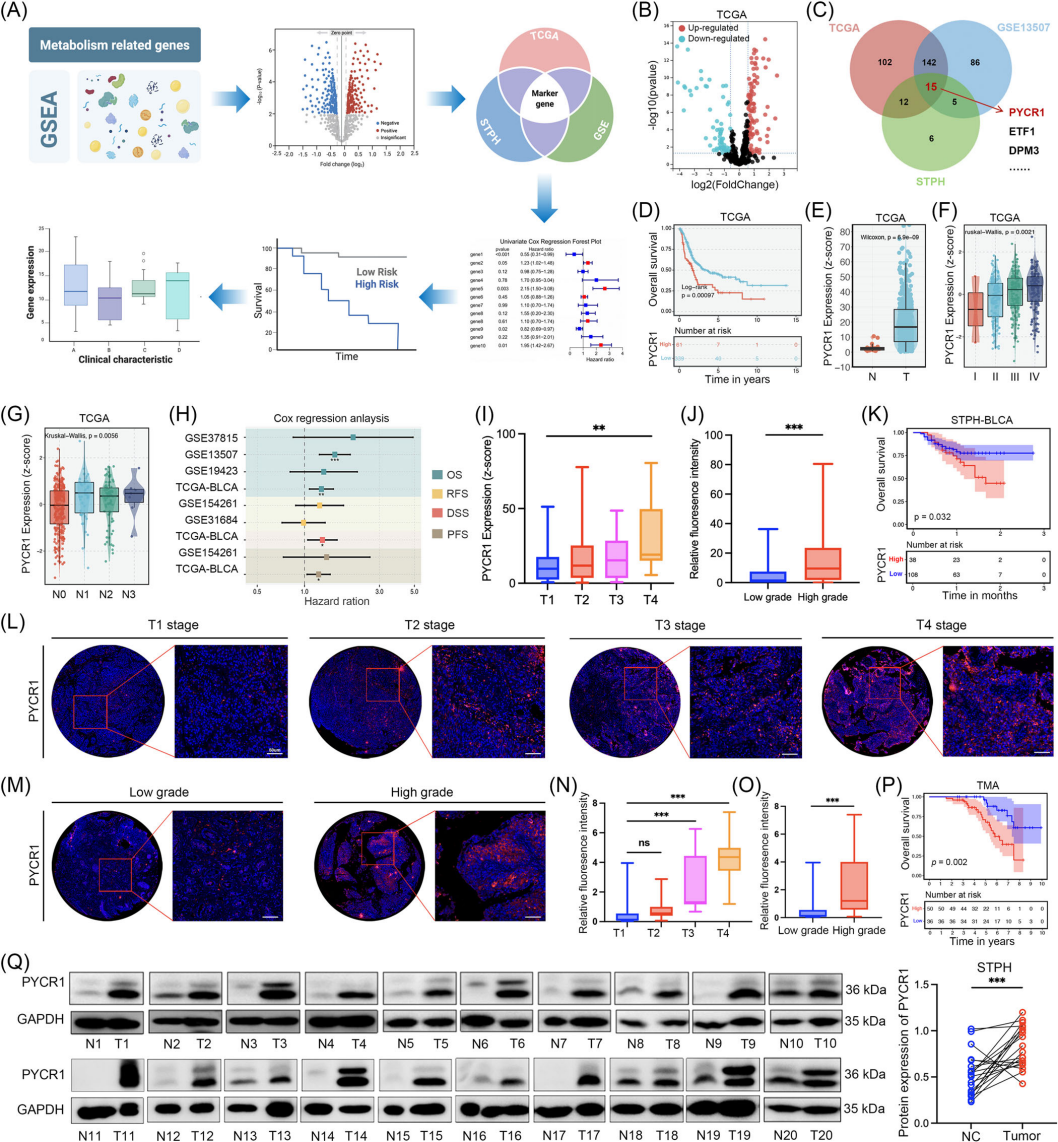
PYCR1 exhibited elevated expression in BCa tissues and correlates with unfavourable prognosis. (A) Schematic diagram of the PYCR1 screening workflow. (B) Differential gene expression profiling of BCa versus normal tissues in the TCGA cohorts. (C) The Venn diagram identified 15 overlapping genes among the three datasets, including PYCR1. (D) Kaplan‑Meier (KM) survival analysis demonstrated that patients with high PYCR1 expression exhibited poor prognosis. (E) PYCR1 exhibited significantly elevated expression in tumour tissues in the TCGA cohort. (F, G) Clinicopathological correlation analysis revealed that high PYCR1 expression was associated with advanced overall pathological stage and lymph node metastasis. (H) Meta‐analysis of multiple BCa datasets revealed that high PYCR1 expression correlates with poor prognosis. (I,J) PYCR1 was upregulated in tumours and correlated with advanced T stage and tumour grade in the STPH cohort. (K) High PYCR1 expression indicated poor prognosis in the STPH cohort. (L, M) Representative IF images of PYCR1 staining of BCa tissue microarrays (TMAs). Scale bar = 50 µm. (N–P) IF intensity of PYCR1 correlated with advanced T stage, grade and unfavourable prognosis. (Q) Western blot analysis of PYCR1 expression in tumor tissues and paired adjacent normal tissues. Each bar represents the mean ± SD, and the *p*‐value was determined using Student's *t*‐test. *p < 0.05, **p < 0.01 and ***p < 0.001.

The panel of metabolism‐associated genes was derived from GSEA, and Figure 1A illustrates the PYCR1 screening process. DEGs between tumour and adjacent normal tissues were analysed using the STPH, TCGA and GSE13507 datasets (Figure 1B and Figure S1A,B). A total of 15 overlapping genes were obtained via the intersection of DEGs and metabolism‐related genes (Figure 1C). Univariate Cox regression analysis identified five genes with prognostic significance, among which PYCR1 was the most prominent prognostic gene (Figure S1C). Prognostic analysis based on the TCGA database demonstrated that high expression of PYCR1 was significantly associated with poor prognosis (Figure 1D). Clinicopathological analyses revealed that PYCR1 was significantly upregulated in BCa tissues (Figure 1E) and that elevated PYCR1 expression was significantly associated with advanced pathological stage, N stage, M stage, and tumour grade (Figure 1F,G and Figure S1D,E). Similar findings were also validated in the GSE13507 cohort (Figure S1G–K). Meta‐analysis demonstrated that PYCR1 was significantly associated with poor prognosis in patients from multiple public BCa datasets (Figure 1H). Moreover, we confirmed the expression of PYCR1 and its correlation with unfavourable prognosis in the STPH cohort, suggesting that PYCR1 might contribute to the malignant progression of BCa (Figure 1I–K). IF analysis of BCa tissue microarrays (TMAs) demonstrated that PYCR1 expression intensity was significantly positively correlated with pathological T stage and histological grade (Figure 1L–O). Notably, patients with high PYCR1 expression had a significantly poorer prognosis (Figure 1P). The clinicopathological characteristics of the patients included in the TMAs are summarised in Table S4. Western blot assays demonstrated that PYCR1 expression was significantly elevated in tumour tissues compared with their paired adjacent normal counterparts, based on specimens from 20 BCa patients (Figure 1Q). Additionally, univariate Cox regression analysis revealed that elevated PYCR1 expression constitutes a significant risk factor for BCa (Figure S1L).

### 3.2 PYCR1 facilitates the proliferation and migration of BCa in vitro

**FIGURE 2:**
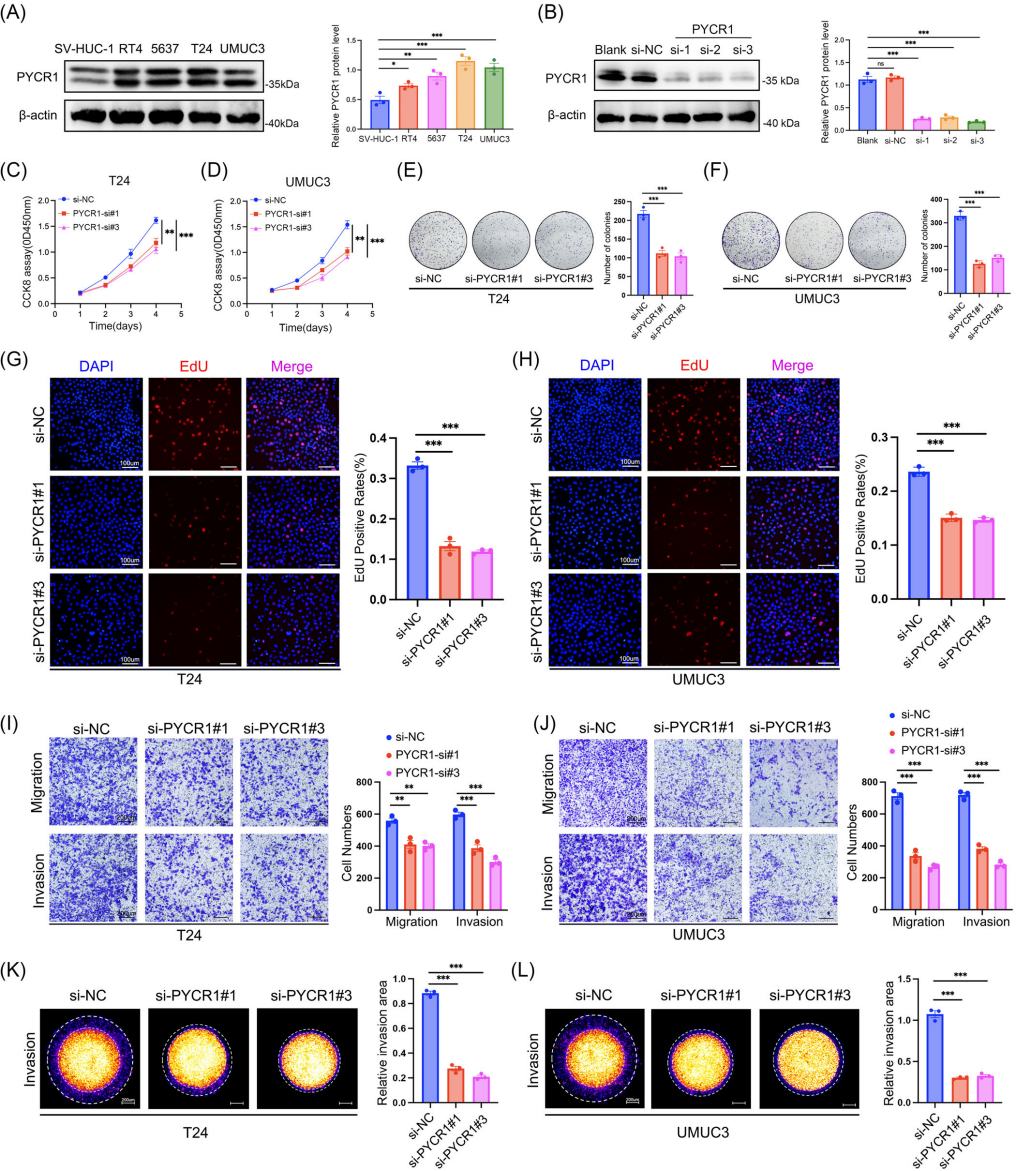
PYCR1 facilitated the proliferation, migration and invasion of BCa in vitro. (A) Western blot was performed to validate PYCR1 expression in normal urothelial cell lines and various BCa cell lines. (B) Western blot confirmed the silencing efficiency of PYCR1‐targeting small interfering RNA (siRNA). (C–H) CCK‑8, colony formation, and EdU assays were performed in T24 and UMUC3 cells to assess the effect of PYCR1 knockdown on BCa cell proliferation. Scale bar = 100 µm. (I, J) Transwell analysis assessed migration and invasion of BCa cells following PYCR1 knockdown. Scale bar = 200 µm. (K, L) 3D Transwell invasion assays were performed to determine the invasive capacity of BCa cells after PYCR1 knockdown. Scale bar = 200 µm. Each bar represents the mean ± SD (*n* = 3). *p*‐Value was calculated using Student's *t*‐test. *p < 0.05, **p < 0.01 and ***p < 0.001.

Quantitative polymerase chain reaction (qPCR) and Western blot assays were performed to detect PYCR1 expression in various BCa cell lines and normal urothelial cells. Results demonstrated that PYCR1 expression was significantly elevated in all tested BCa cell lines relative to normal controls (Figure 2A and Figure S2A). To elucidate the role of PYCR1 in BCa, we knocked down PYCR1 in T24 cells and subsequently performed a series of functional assays. The efficiency of PYCR1 knockdown was confirmed at both the protein and mRNA levels by Western blot and qPCR, respectively (Figure 2B and Figure S2B). Cell counting kit‐8 (CCK‐8), colony formation, and EdU assays demonstrated that PYCR1 knockdown significantly suppressed the proliferative capacity of T24 and UMUC3 BCa cells (Figure 2C–H). In scratch wound healing assays, PYCR1 knockdown resulted in marked retardation of wound closure, indicative of impaired cell migration (Figure S2C,D). Consistently, Transwell assays revealed that PYCR1 knockdown markedly reduced the invasive penetration of BCa cells (Figure 2I,J), and this inhibitory effect on cell invasion was further corroborated by 3D Matrigel invasion assays (Figure 2K,L). Furthermore, Western blot assays verified that PYCR1 knockdown altered the expression of epithelial–mesenchymal transition (EMT)‐related biomarkers, including N‐cadherin, Vimentin, and E‐cadherin (Figure S2E). Clinical nomograms constructed based on PYCR1 expression exhibited robust efficacy in predicting patient prognosis (Figure S2F–H). Collectively, these in vitro assays demonstrated that PYCR1 promotes the proliferation and migration of BCa cells.

### 3.3 PYCR1 promotes the progression and metastasis of BCa in vivo

**FIGURE 3:**
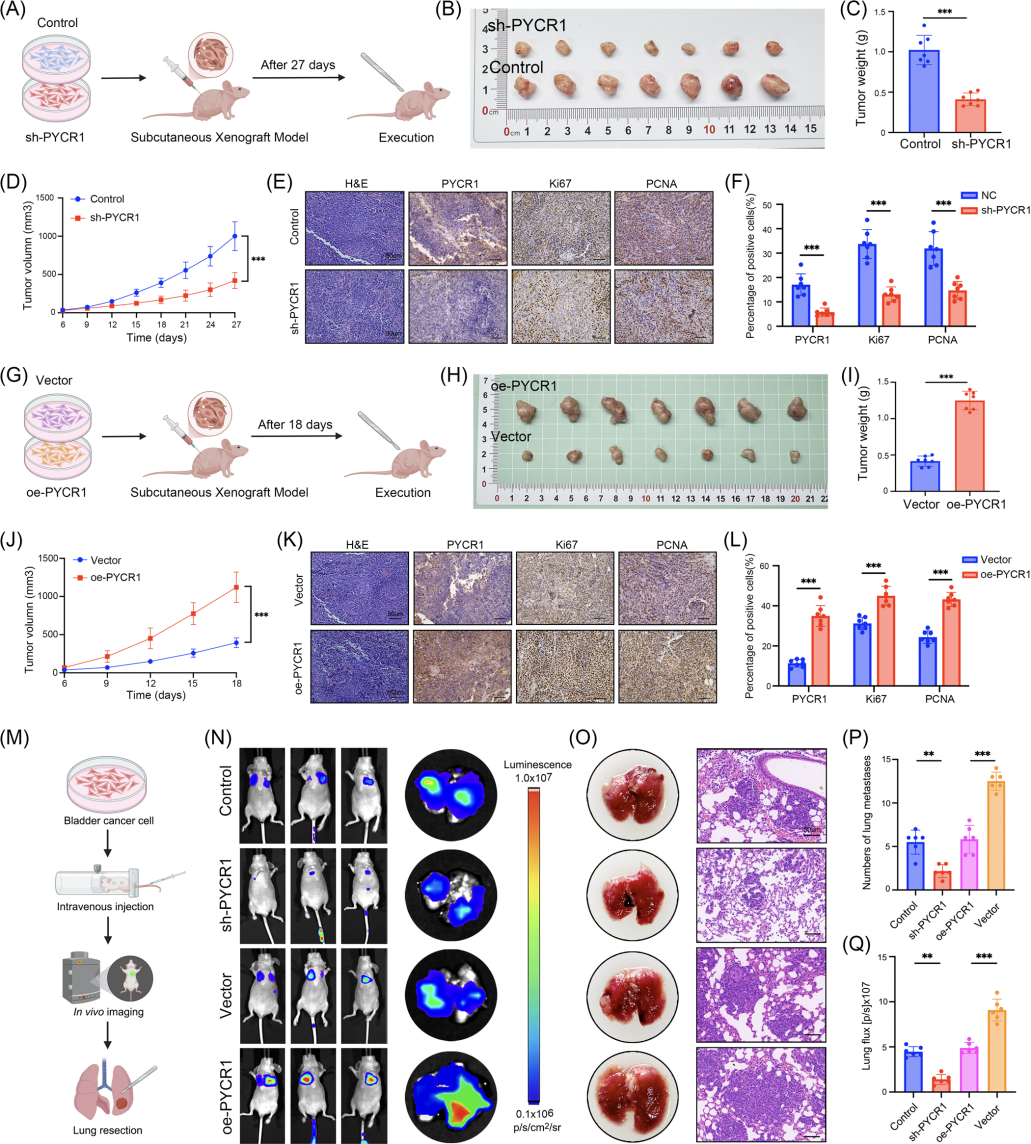
PYCR1 promoted the progression and metastasis of BCa in vivo. (A, G) Schematic diagram of subcutaneous tumour model establishment with PYCR1 knockdown or overexpression. (B, H) Bright‐field images of resected subcutaneous tumours in PYCR1 knockdown or overexpression groups (*n* = 7). (C, D, I, J) Quantitative analysis of tumour weight and volume following PYCR1 knockdown or overexpression. (E‐F, K‐L) Representative IHC staining images of PYCR1, Ki67 and PCNA in tumour tissues, along with H&E staining images. Scale bar = 50 µm. (M) Schematic diagram of lung metastasis model establishment via tail vein injection. (N) Representative bioluminescence imaging of lung metastases (*n* = 6). (O) Representative bright‐field images and H&E staining images of lung metastatic tumour tissues. Scale bar = 50 µm. (P, Q) Quantitative analysis of lung metastatic tumour number and fluorescence intensity. Data were presented as means ± SD, and *p*‐values were determined using Student's *t*‐test. *p < 0.05, **p < 0.01 and ***p < 0.001.

The effects of PYCR1 knockdown or overexpression on tumour proliferation and metastasis were evaluated in vivo using subcutaneous xenograft tumour and tail vein injection‐induced lung metastasis models. Stable PYCR1 knockdown and overexpression in T24 and UMUC3 cell lines were established, and the transfection efficiency was validated by qPCR and Western blot (Figure S3A–D). In the subcutaneous xenograft model, PYCR1 knockdown significantly suppressed tumour volume and weight, whereas its overexpression markedly promoted tumour growth (Figure 3A–D and Figure 3G–J). Haematoxylin and eosin (H&E) staining confirmed that PYCR1 knockdown reduced tumour malignancy, whereas its overexpression enhanced this malignant phenotype. Immunohistochemical (IHC) analysis revealed that the expression of proliferation markers Ki‐67 and PCNA was downregulated in PYCR1‐knockdown tumours and upregulated in PYCR1‐overexpressing tumours, respectively (Figure 3E,F and Figure 3K,L). In the lung metastasis model, pulmonary luciferase signal intensity was significantly reduced in the PYCR1 knockdown group, whereas it was markedly enhanced in the overexpression group (Figure 3M,N,Q). H&E staining of lung tissues revealed that PYCR1 knockdown mice exhibited fewer metastatic nodules, whereas PYCR1 overexpression mice developed a markedly higher number of metastatic lesions (Figure 3O,P). These in vivo results confirm that PYCR1 plays an essential role in mediating BCa proliferation and metastasis.

### 3.4 PYCR1 upregulates APOE to modulate JAK2/STAT3 signalling pathway

**FIGURE 4:**
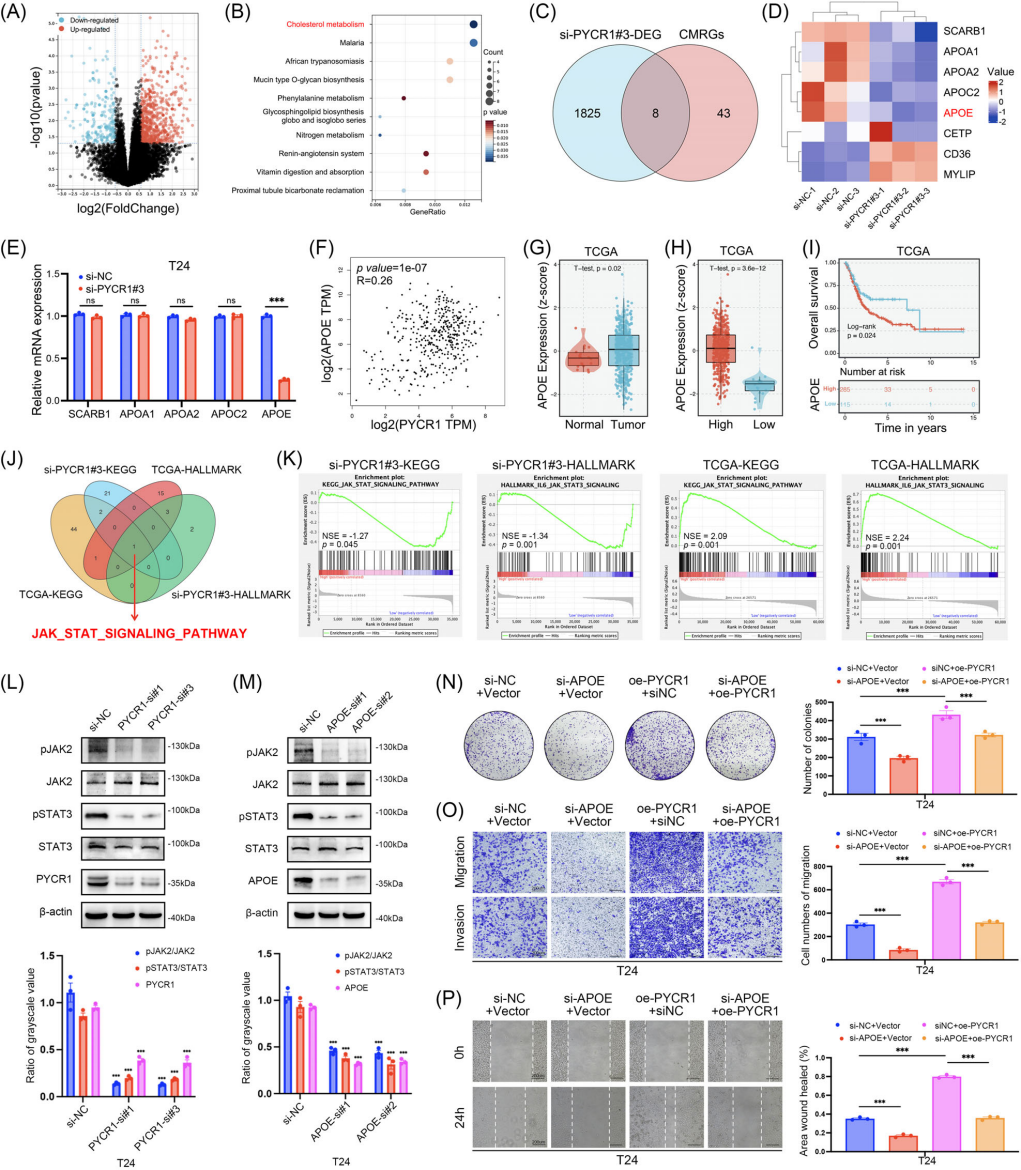
PYCR1 upregulated APOE to modulate the JAK2/STAT3 signalling pathway. (A) Volcano plots showed differentially expressed genes (DEGs) between the control and PYCR1 knockdown groups (*n* = 3). (B) KEGG analysis highlighting the main signalling pathways enriched in DEGs. (C) Venn diagrams showed the intersection genes between DEGs and cholesterol metabolism‐related genes (CMRGs). (D) Heatmaps revealed the expression of overlapping genes in different samples. (E) qPCR validation of mRNA levels of CMRGs following PYCR1 knockdown. (F) Correlation analysis between PYCR1 and APOE using the TCGA database. (G, H) PYCR1 was highly expressed in tumour tissues, especially in high‐grade BCa. (I) Patients with high APOE expression exhibited poor prognosis. (J, K) GSEA was performed on PYCR1 knockdown RNA‐seq data and TCGA data with KEGG and Hallmark databases, respectively. (L, M) Western blot analysis of JAK2/STAT3 pathway‐related protein expression following PYCR1 or APOE knockdown. (N) Colony formation assays were performed to assess whether PYCR1 overexpression could rescue the proliferation suppressed by APOE knockdown in T24 cells. (O, P) Transwell and wound healing assays were conducted to evaluate whether PYCR1 overexpression could reverse the impaired invasion and migration induced by APOE knockdown. Scale bar = 200 µm. Data were presented as means ± SD (*n* = 3), and *p*‐values were determined using Student's *t*‐test. *p < 0.05, **p < 0.01 and ***p < 0.001.

To explore the downstream regulatory molecules of PYCR1, transcriptome sequencing was performed on PYCR1 knockdown T24 cells. Volcano plot analysis identified 1833 DEGs between PYCR1 knockdown T24 cells and the control group, among which 1295 were significantly upregulated, and 538 were significantly downregulated (Figure 4A). KEGG pathway enrichment analysis of the identified DEGs revealed that the cholesterol metabolism pathway was the most significantly enriched (Figure 4B). Subsequently, we intersected the DEGs with cholesterol metabolism‐related genes (CMRGs), identifying 8 overlapping genes (Figure 4C and Table S5). A heatmap was generated to visualise the expression profiles of these overlapping genes (Figure 4D). qPCR was performed to validate the expression of downregulated candidate genes (SCARB1, APOA1, APOA2, APOC2 and APOE) in the si‐PYCR1 group in T24 and UMUC3 cell lines, and the results demonstrated that only APOE expression was markedly reduced (Figure 4E and Figure S4A). Correlation analyses of PYCR1 and the candidate genes using TCGA data revealed that APOE exhibited the strongest correlation with PYCR1, providing independent support for our experimental findings (Figure 4F and Figure S4B). Clinical analyses demonstrated that APOE was highly expressed in BCa tissues and correlated with unfavourable clinicopathological characteristics and poor prognosis (Figure 4G–I). Using data from si‐PYCR1 knockdown cells and the TCGA dataset, we performed GSEA with the KEGG and Hallmark databases, respectively; the results revealed that the JAK/STAT signalling pathway was the sole overlapping pathway (Figure 4J,K). Western blots were performed to investigate the effects of PYCR1 and APOE on the expression of proteins associated with the IL‐6/JAK/STAT3 signalling pathway; the results demonstrated that knockdown of PYCR1 and APOE significantly reduced the protein expression levels of phosphorylated JAK2 (p‐JAK2) and phosphorylated STAT3 (p‐STAT3), whereas their total protein levels remained largely unchanged (Figure 4L,M). The efficiency of APOE knockdown was confirmed by qPCR and Western blot analyses (Figure S4C–F). These results suggest that PYCR1 may regulate the JAK2/STAT3 signalling pathway through APOE. Colony formation, Transwell, and scratch wound healing assays demonstrated that APOE knockdown could reverse the promotion of BCa cell proliferation and migration induced by PYCR1 overexpression (Figure 4N–P).

### 3.5 PYCR1 interacts with NUDT5 to enhance ATP production

**FIGURE 5:**
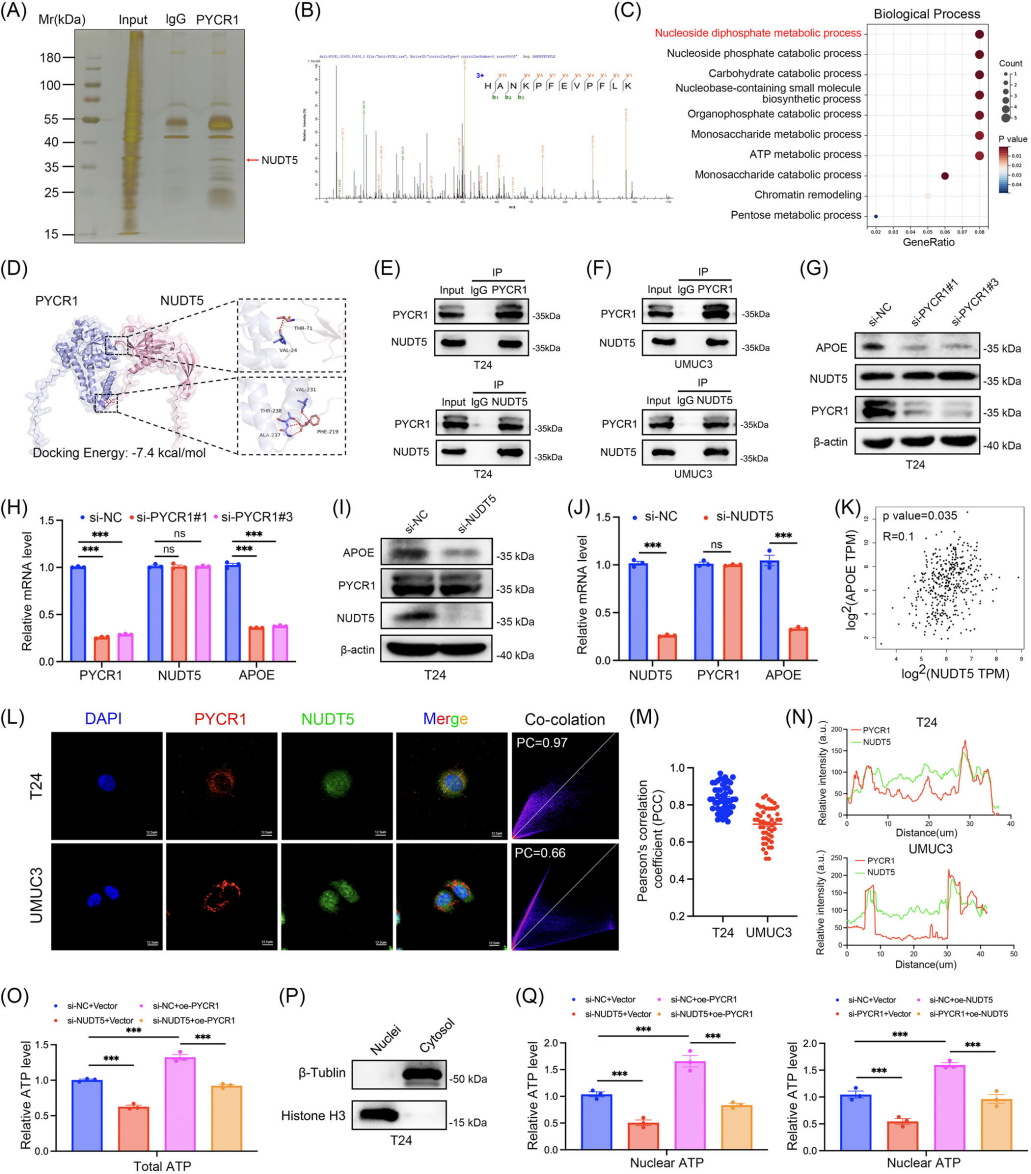
PYCR1 interacted with NUDT5 to enhance adenosine triphosphate (ATP) production. (A) Co‐immunoprecipitation (Co‐IP) combined with silver staining was employed to identify the interacting proteins of PYCR1. (B) Mass spectrometry analysis revealed NUDT5 as an interacting protein of PYCR1. (C) KEGG pathway enrichment analysis was performed on the interacting proteins of PYCR1. (D) 3D molecular docking simulation of the interaction between PYCR1 and NUDT5. (E, F) Co‐immunoprecipitation assays confirmed the endogenous interaction between PYCR1 and NUDT5 in T24 and UMUC3 cells. (G) Western blot was performed to assess the protein expression of APOE and NUDT5 following PYCR1 knockdown. (H) qPCR was performed to evaluate the mRNA expression of NUDT5 and APOE following PYCR1 knockdown. (I) Western blot was conducted to evaluate the protein expression of APOE and PYCR1 after NUDT5 knockdown. (J) qPCR was performed to verify the APOE and PYCR1 mRNA levels following NUDT5 knockdown. (K) Correlation analysis between NUDT5 and APOE based on the TCGA database. (L) Representative immunofluorescence (IF) images showing the localisation of PYCR1 and NUDT5. Scale bar = 12.5 µm. (M) Quantification of the Pearson correlation coefficient in 50 cells per cell line. (N) Quantitative analysis of PYCR1 and NUDT5 IF intensity. (O) ATP levels were measured to assess whether PYCR1 overexpression restored total ATP production following NUDT5 knockdown in T24 cells. (P) Western blot was performed to verify the purity of the nuclear and cytoplasmic fractions. (Q) Quantification of relative nuclear ATP levels shows that NUDT5 knockdown attenuates the increase induced by PYCR1 overexpression, whereas NUDT5 overexpression partially rescues the reduction caused by PYCR1 knockdown. Each bar represents the mean ± SD (*n* = 3). *p*‐Values were calculated using Student's *t*‐test. *p < 0.05, **p < 0.01 and ***p < 0.001.

To elucidate the molecular mechanism underlying PYCR1‐mediated regulation of APOE, we identified potential interacting substrates via co‐immunoprecipitation (Co‐IP) coupled with silver staining and mass spectrometry (MS) (Figure 5A). MS analysis identified NUDT5 as a key binding protein of PYCR1. Specifically, NUDT5 exhibited a sequence coverage of 19% with a Sequest HT score of 13.78, and four unique peptides were detected. Moreover, the observed molecular weight of NUDT5 was 24.30 kDa, which was consistent with its theoretical molecular weight of 23.90 kDa (UniProt ID: Q9UKK9), thus validating the reliability of the MS results (Figure 5B). KEGG pathway enrichment analysis revealed that the nucleoside diphosphate metabolic process was the most significantly enriched pathway (Figure 5C). Molecular docking simulations predicted the structural and molecular basis of the PYCR1–NUDT5 interaction, with an estimated binding energy of −7.4 kcal/mol (Figure 5D). Endogenous Co‐IP assays confirmed the interaction between PYCR1 and NUDT5 in both T24 and UMUC3 cell lines (Figure 5E,F). Moreover, exogenous Co‐IP assays were conducted in 293T cells co‐expressing FLAG‐tagged PYCR1 and HA‐tagged NUDT5 (Figure S5A). These experiments consistently demonstrated a reproducible interaction between PYCR1 and NUDT5 across different cellular contexts. Western blot and qPCR assays were performed to verify that PYCR1 knockdown reduced the protein and mRNA expression of APOE, while exerting no significant effect on NUDT5 expression (Figure 5G,H). Consistently, NUDT5 knockdown reduced both APOE protein and mRNA levels, while PYCR1 expression remained unaffected (Figure 5I,J). The knockdown and overexpression efficiencies of NUDT5 are shown in Figure S5B,C. The rescue experiments further demonstrated that NUDT5 overexpression restored APOE mRNA and protein levels in PYCR1 knockdown cells (Figure S5D,E). Moreover, PYCR1 overexpression significantly increased APOE expression, whereas NUDT5 knockdown markedly attenuated this effect (Figure S5F,G). In addition, using a catalytically inactive NUDT5 mutant (NUDT5‐Mut), we demonstrated that loss of NUDT5 enzymatic activity abolished its ability to rescue the reduction in APOE protein and mRNA expression caused by PYCR1 knockdown (Figure S5H,I). These results suggest that PYCR1 may upregulate APOE expression by modulating the functional activity of NUDT5, rather than by altering its expression. NUDT5 mRNA expression was positively correlated with APOE expression in TCGA cohorts (Figure 5K). IF staining assays demonstrated that PYCR1 and NUDT5 exhibited significant spatial colocalization (Figure 5L). Quantitative colocalization analysis using the Pearson correlation coefficient (PCC) demonstrated that PYCR1 and NUDT5 exhibited a robust positive correlation across both BCa cell lines (Figure 5M). Line profile analysis further corroborated that the fluorescence intensity peaks of PYCR1 and NUDT5 were aligned with the analysed dashed lines (Figure 5N). Total cellular ATP levels were quantified using an ATP assay kit. PYCR1 overexpression significantly increased intracellular ATP levels, whereas NUDT5 knockdown markedly attenuated this increase (Figure 5O). Nuclear–cytoplasmic fractionation was subsequently performed, and the purity of the nuclear and cytoplasmic fractions was verified by Western blot (Figure 5P). Furthermore, rescue experiments demonstrated that PYCR1 overexpression significantly increased nuclear ATP levels, whereas NUDT5 knockdown markedly attenuated this increase. Conversely, PYCR1 knockdown significantly reduced nuclear ATP levels, while NUDT5 overexpression partially rescued the reduction caused by PYCR1 depletion (Figure 5Q).

To exclude the potential contribution of PYCR1 enzymatic activity to downstream pathway regulation, we used the selective PYCR1 inhibitor PYCR1‐IN‐1 to suppress its catalytic activity. Intracellular proline levels were measured using a proline assay kit and were significantly reduced in T24 cells following PYCR1‐IN‐1 treatment (Figure S5J). Western blot analysis showed that PYCR1‑IN‑1 treatment did not affect PYCR1 protein abundance. Moreover, it had no significant impact on NUDT5 and APOE protein levels, nor on JAK2/STAT3 phosphorylation (Figure S5K). Colony formation and Transwell assays showed that, although PYCR1‐IN‐1 exerted modest inhibitory effects on cell proliferation and migration, PYCR1 overexpression significantly restored the proliferative and migratory capacities of T24 cells subjected to combined PYCR1 knockdown and PYCR1‐IN‐1 treatment (Figure S5L,M). These findings support an enzyme activity‐independent role of PYCR1 in promoting malignant phenotypes. Multiplex IF staining was performed to examine PYCR1, NUDT5, and APOE expression in the same BCa tissue sections. PYCR1 and NUDT5 exhibited spatial overlap within the tumour tissue, supporting their close association at the tissue level. In contrast, APOE displayed a more diffuse staining pattern within the same tumour regions, consistent with its secreted nature (Figure S5N).

### 3.6 NUDT5‐dependent ATP production sustains SP1‐mediated APOE transcription

Potential APOE transcription factors were screened using multiple prediction databases, including JASPAR, TFBS, GTRD and ChIP‐Atlas. SP1, ZNF281 and ZBTB7A were identified as overlapping candidate transcription factors (Figure S6A). Analysis of publicly available ChIP‐seq datasets revealed prominent SP1 enrichment peaks within the APOE promoter region in H1 embryonic stem cells, A549 lung cancer cells, and HepG2 hepatocellular carcinoma cells (Figure S6B). Previous studies have identified functional SP1‐responsive elements within the proximal promoter of human APOE and demonstrated that SP1 enhances APOE transcriptional activity.^32^, ^33^ Western blot and qPCR analyses demonstrated that SP1 knockdown significantly reduced APOE mRNA and protein levels (Figure S6C,D). Potential SP1‐binding motifs within the APOE promoter were subsequently predicted using JASPAR (Figure S6E), and reporter constructs containing either the wild‐type APOE promoter or a mutant promoter with disruption of the predicted SP1‐binding site were generated (Figure S6F). Dual‐luciferase reporter assays showed that the wild‐type APOE promoter exhibited strong transcriptional activity, whereas mutation of the SP1‐binding site significantly impaired promoter activity. Notably, oligomycin (an ATP synthase inhibitor) treatment significantly reduced the transcriptional activity of the APOE‐WT promoter, indicating that SP1‐mediated APOE transcription requires sufficient ATP availability (Figure S6G). Oligomycin treatment significantly reduced nuclear ATP levels in the T24 cell line (Figure S6H). Western blot analysis further showed that oligomycin treatment reduced APOE protein expression and decreased the phosphorylation levels of JAK2 and STAT3, whereas the total protein levels of JAK2 and STAT3 remained largely unchanged. Notably, oligomycin treatment did not significantly alter SP1 protein abundance (Figure S6I).

Colony formation and Transwell assays further demonstrated that APOE overexpression effectively rescued the impaired proliferative and migratory capacities caused by NUDT5 knockdown (Figure S6J,K). Western blot showed that NUDT5 knockdown suppressed the activation of the downstream JAK2/STAT3 signalling pathway, whereas APOE overexpression reversed this inhibitory effect (Figure S6L).

### 3.7 PYCR1 promotes BCa progression via NUDT5

**FIGURE 6:**
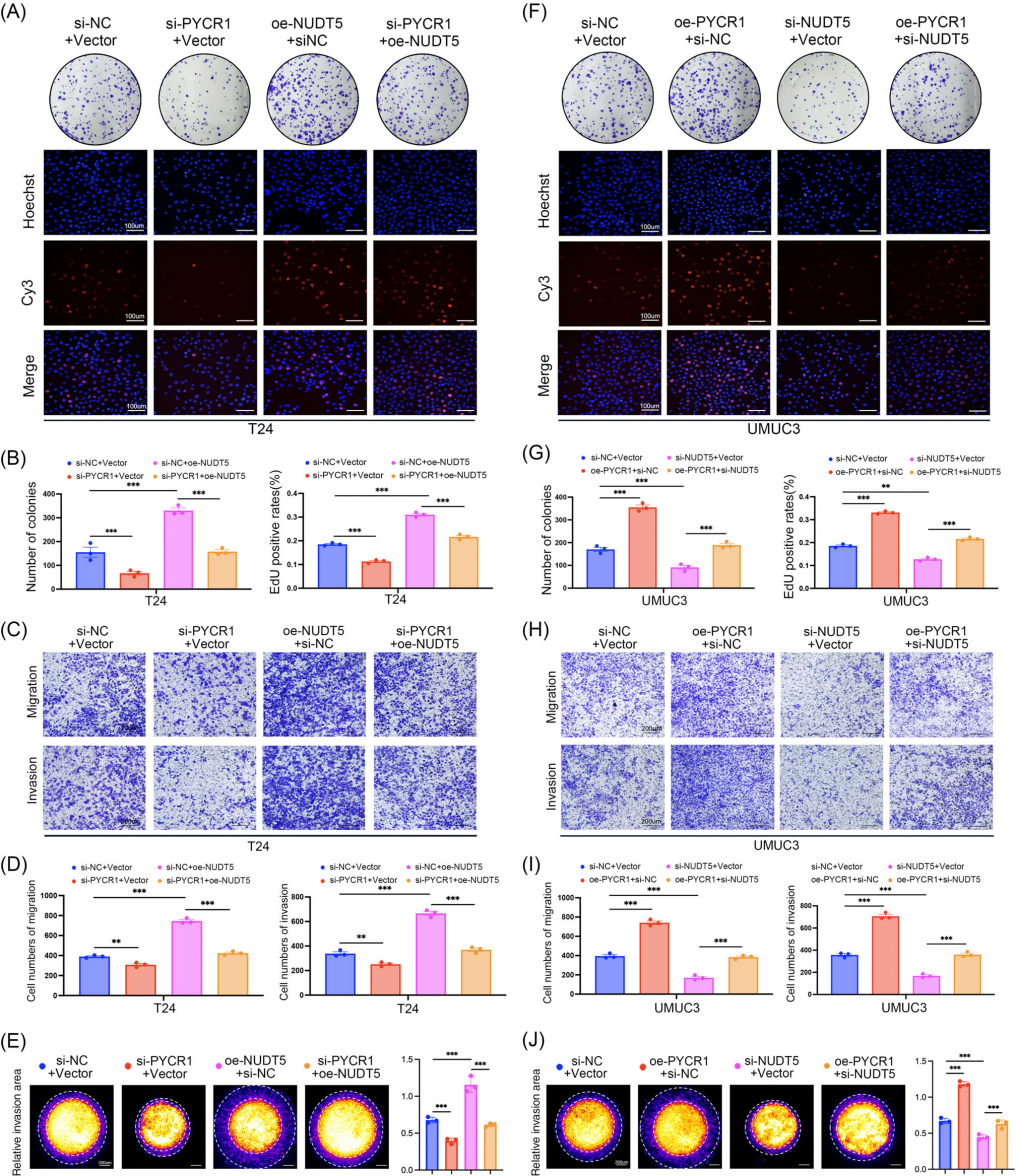
PYCR1 promoted BCa proliferation, migration and invasion via NUDT5. (A, B). Colony formation and EdU assays demonstrate that NUDT5 overexpression rescues the impaired proliferative capacity caused by PYCR1 knockdown. Bar graphs show the corresponding quantitative results. Scale bar = 100 µm. (C–E) Transwell and 3D Transwell invasion assays demonstrate that NUDT5 overexpression rescues the impaired migratory and invasive capacities caused by PYCR1 knockdown. Bar graphs show the corresponding quantitative results. Scale bar = 200 µm. (F, G) Colony formation and EdU assays demonstrate that NUDT5 knockdown suppresses the enhanced proliferative capacity induced by PYCR1 overexpression, with the corresponding quantitative results presented as bar graphs. Scale bar = 100 µm. (H–J) Transwell and 3D Transwell invasion assays demonstrate that NUDT5 knockdown reverses the enhanced migratory and invasive capacities induced by PYCR1 overexpression, with the corresponding quantitative results presented as bar graphs. Scale bar = 200 µm. Each bar represents the mean ± SD (*n* = 3). *p*‐Values were calculated using Student's *t*‐test. *p < 0.05, **p < 0.01, ***p < 0.001.

To further elucidate the role of NUDT5 in PYCR1‐driven tumour progression, we performed rescue experiments. Colony formation and EdU assays showed that PYCR1 knockdown inhibited the proliferation of T24 cells, whereas NUDT5 overexpression reversed this inhibitory effect. (Figure 6A,B). Transwell, wound healing, and 3D Transwell assays demonstrated that NUDT5 overexpression rescued the impaired migration and invasion capacities of T24 cells caused by PYCR1 knockdown (Figure 6C–E and Figure 6K). Furthermore, similar functional assays in UMUC3 cells demonstrated that NUDT5 knockdown reversed the enhanced proliferation, migration, and invasion capacities induced by PYCR1 overexpression (Figure 6F–J and Figure S6L). Collectively, these reciprocal rescue experiments demonstrate that NUDT5 is a critical downstream mediator of PYCR1‐driven proliferation, migration, and invasion in BCa cells.

### 3.8 TH5427 suppresses BCa progression by targeting NUDT5 in vitro and in vivo

**FIGURE 7:**
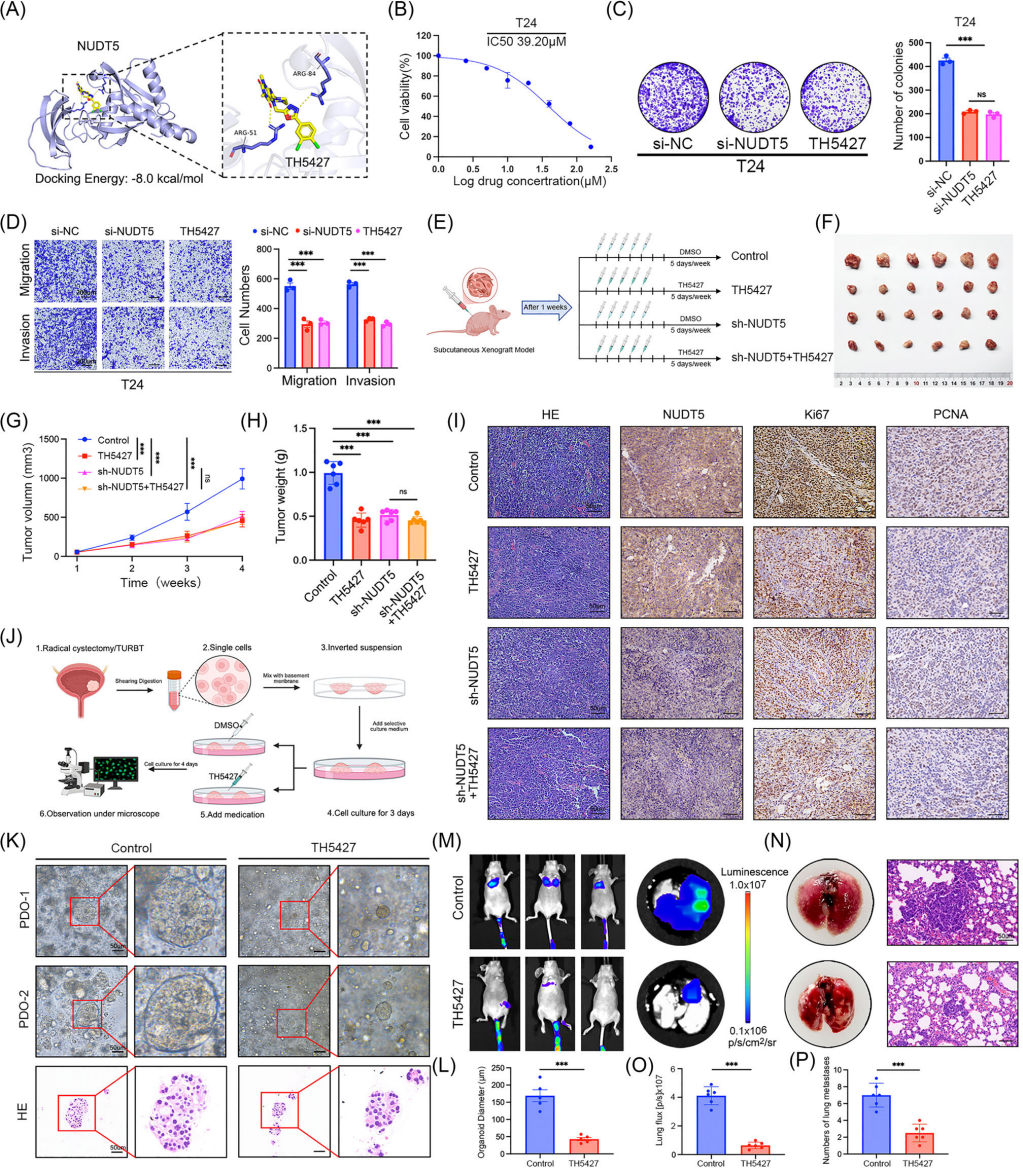
TH5427 suppressed BCa progression by targeting NUDT5 in vitro and in vivo. (A) 3D docking model illustrating the interaction between the NUDT5 protein and the inhibitor TH5427. (B) The IC50 of T24 cells following TH5427 treatment was determined using the CCK‐8 assay. (C) Colony formation assays were performed to evaluate the proliferative capacity of cells in the si‐NC, si‐NUDT5 and TH5427‐treated groups. (D) Transwell assays were performed to assess cell migration and invasion in the indicated groups. Scale bar = 200 µm. (E) Schematic illustration of the subcutaneous xenograft tumour model with TH5427 treatment and/or NUDT5 knockdown. (F–H) Bright‐Field images of the excised tumours and quantitative analyses of tumour weight and volume (*n* = 6). (I) Representative H&E staining of tumour tissues and IHC staining for NUDT5, Ki67, and PCNA. (J) Flowchart of BCa patient‐derived organoid establishment (PDOs). (K, L) Representative bright‐field and H&E staining images of PDOs with corresponding quantification of organoid diameter (*n* = 5). Scale bar = 50 µm. (M‐N) The inhibitory effect of TH5427 on BCa metastasis was evaluated using the lung metastasis model (*n* = 6). Scale bar = 50 µm. (O, P) Quantification of fluorescence intensity and the number of lung metastases in Control and TH5427‐treated groups. Each bar represents the mean ± SD (*n* = 3). *p*‐Values were calculated using Student's *t*‐test. *p < 0.05, **p < 0.01 and ***p < 0.001.

**FIGURE 8:**
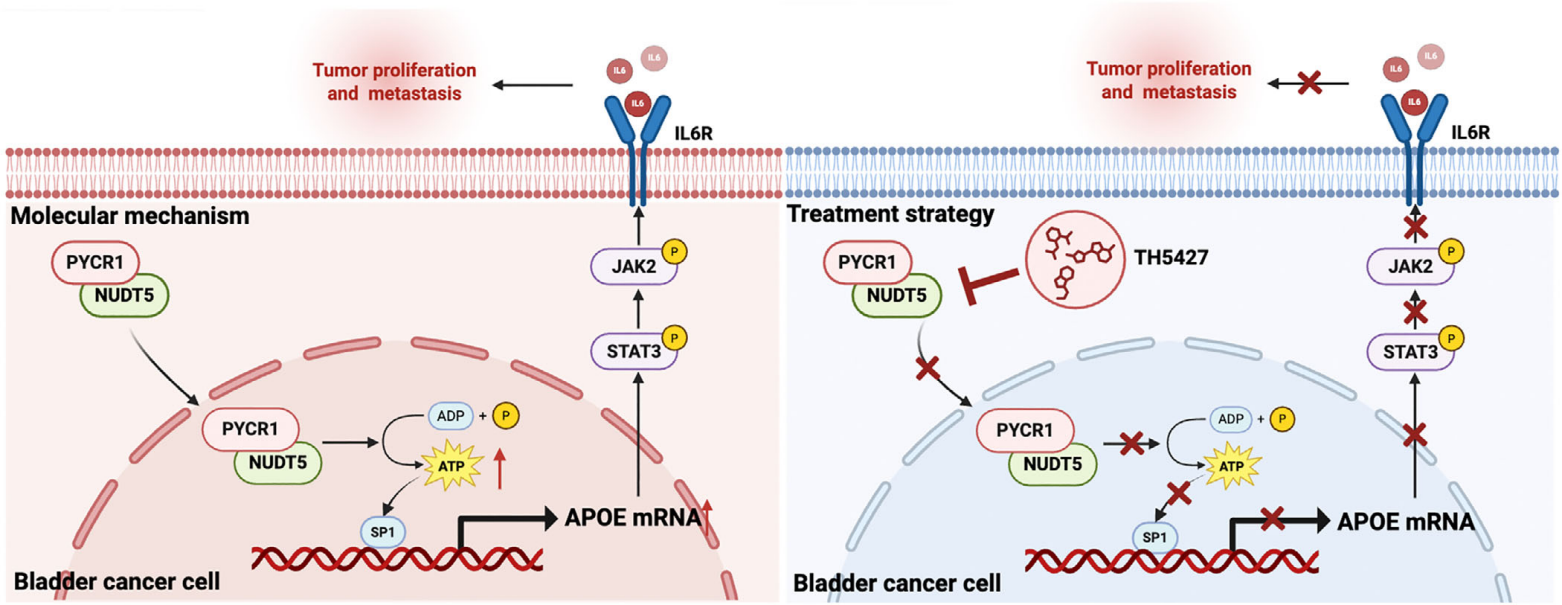
Schematic illustration of the proposed mechanism. PYCR1, via NUDT5‑dependent nuclear adenosine triphosphate (ATP) production, promotes SP1‑mediated APOE transcription. APOE, in turn, activates the JAK2/STAT3 signalling pathway, thereby promoting the proliferative and metastatic capacities of BCa cells. TH5427, a small‐molecule inhibitor targeting NUDT5 enzymatic activity, effectively counteracts this process.

Collectively, these findings underscore the critical role of NUDT5 in BCa progression, thereby implicating NUDT5‐targeted agents as a promising therapeutic strategy. TH5427 has been identified as a highly effective NUDT5 inhibitor that can effectively block nuclear ATP synthesis, thereby inhibiting chromatin remodelling, gene regulation, and proliferation. Molecular docking assays confirmed that TH5427 forms stable binding interactions with NUDT5 (Docking Energy: ‐8.0 kcal/mol, Figure 7A). CCK‐8 assay results demonstrated that TH5427 markedly inhibited the proliferation of T24 (IC_50_ = 39.20 µM) and UMUC3 (IC_50_ = 31.28 µM) cells (Figure 7B and Figure S7A). Colony formation and Transwell assays showed that TH5427 (30 µM) significantly suppressed the proliferation, migration, and invasion of BCa cells (Figure 7C,D and Figure S7B–E). Furthermore, ATP quantification assays revealed that TH5427 suppressed intracellular ATP production (Figure S7F). To investigate the effect of TH5427 on NUDT5 expression, T24 and UMUC3 cells were treated with increasing concentrations of TH5427 (0, 20, 40 and 80 µM) for 48 h. Western blot showed that TH5427 treatment did not significantly alter NUDT5 protein levels (Figure S7G). Importantly, TH5427 suppressed the activation of the JAK2/STAT3 signalling pathway (Figure S7H). Subcutaneous xenograft tumour models were employed to assess the in vivo efficacy of TH5427. Tumour‐bearing mice were randomly assigned to four groups: control, TH5427, sh‐NUDT5, and sh‐NUDT5+TH5427. According to group allocation, the mice received intraperitoneal injections of either TH5427 (50 mg/kg) or vehicle (DMSO) on the same treatment schedule (Figure 7E). Specifically, TH5427 treatment was initiated 1 week after tumour cell inoculation. TH5427 was administered for 5 consecutive days per week followed by a 2‐day treatment‐free interval, and this treatment schedule was continued for a total of 3 weeks. Tumour volumes were measured weekly throughout the treatment period. The results showed that both NUDT5 knockdown and TH5427 treatment significantly suppressed tumour growth. Importantly, TH5427 treatment in mice bearing NUDT5‐knockdown tumours did not further significantly suppress tumour growth compared with NUDT5 knockdown alone (Figure 7F–H). IHC analysis of the xenograft tumours showed that TH5427 treatment did not reduce NUDT5 protein expression, whereas the levels of the proliferation markers Ki67 and PCNA were significantly decreased (Figure 7I). To evaluate the biosafety of TH5427 treatment, major organs from the mice, including the heart, liver, spleen, lungs, and kidneys, were histologically examined using H&E staining. The results showed that no apparent pathological abnormalities were observed following TH5427 treatment (Figure S7I). To further validate the clinical translational potential of TH5427, we established PDO models and subjected them to H&E staining (Figure 7J). Results demonstrated that TH5427 significantly suppressed PDO growth (Figure 7K,L). A lung metastasis model was employed to assess the anti‐metastatic potential of TH5427. In vivo imaging and H&E staining consistently demonstrated fewer metastatic nodules and reduced fluorescence intensity in TH5427‐treated mice relative to controls (Figure 7 M–P). In summary, these findings demonstrated that TH5427 exerts a specific inhibitory effect on BCa progression both in vitro and in vivo via targeted suppression of NUDT5. Figure 8 provides a schematic overview of the proposed regulatory mechanism.

## 4 DISCUSSION

Metabolic reprogramming drives the malignant progression of diverse neoplasms.^34^ Elucidating the pivotal metabolic enzymes and regulatory networks governing BCa development, progression and metastasis represents a critical unresolved challenge in basic research, while also holding profound implications for identifying highly specific therapeutic targets and refining clinical treatment strategies. Recent investigations indicate that PYCR1 plays an essential role in proline biosynthesis, while also modulating tumorigenesis through multifaceted mechanisms.^35^, ^36^ Lee and colleagues demonstrated that PYCR1 sustains EGFR stability via assembling a ternary complex with EGFR and USP11, while triggering the TLR signalling pathway, ultimately potentiating lung cancer progression.^37^ In addition, Li and colleagues documented that PYCR1 enhances the transcriptional activity of IRS1 via H3K18 lactylation, thereby facilitating the proliferation and metastatic capacity of hepatocellular carcinoma cells.^38^ A multi‐omics investigation revealed that PYCR1 facilitates proline biosynthesis via P5CS and triggers the PI3K/AKT/mTOR signalling axis, thereby modulating glutamine utilisation and metabolic reprogramming in BCa cells.^39^ This study unveils the non‐canonical oncogenic function of PYCR1 in BCa. PYCR1 activates the JAK2/STAT3 signalling pathway through physical interaction with NUDT5 and upregulates APOE, ultimately leading to the malignant progression of BCa.

To explore the molecular mechanisms underlying PYCR1 function, transcriptome sequencing coupled with KEGG pathway analysis revealed marked enrichment of genes involved in cholesterol metabolism. Although direct evidence that PYCR1 regulates cholesterol metabolism remains limited, accumulating findings suggest a potential link between PYCR1 and cholesterol homeostasis. Stum et al. reported that Pycr2‐deficient mice exhibited a marked reduction in subcutaneous adipose tissue, accompanied by decreased circulating levels of total cholesterol, high‐density lipoprotein cholesterol, and triglycerides.^40^ Given the functional similarity between PYCR1 and PYCR2, these findings raise the possibility that PYCR1 may likewise participate in the regulation of cholesterol metabolism. Consistent with this notion, an integrated mass spectrometry‐based metabolomic and lipidomic analysis of prostate cancer stem cells revealed elevated PYCR1 expression and triglyceride levels in these cells.^41^ APOE is a major lipid transport protein that plays a central role in the transport, cellular uptake, and redistribution of cholesterol and other lipids.^24^ Beyond its canonical function in lipid homeostasis, accumulating evidence indicates that aberrantly expressed APOE can exert context‐dependent tumour‐promoting effects by enhancing cancer cell proliferation, migration, as well as by remodelling the tumour immune microenvironment. For example, tumour‐derived APOE promotes pancreatic neuroendocrine tumour progression by inducing the formation of tip endothelial cells.^42^ APOE can also bind to TREM2 on macrophages, promoting AR expression and inducing an immunosuppressive microenvironment that facilitates prostate cancer progression.^43^ Collectively, these findings suggest that APOE is not merely a marker of altered lipid metabolism but may also function as an important downstream effector of tumour progression. Therefore, elucidating how PYCR1 regulates APOE expression and function is essential for understanding the mechanisms underlying PYCR1‐mediated BCa malignant progression. In this study, Western blot and qPCR demonstrated that PYCR1 knockdown specifically downregulated APOE mRNA and protein levels. The TCGA dataset demonstrated a significant positive correlation between PYCR1 and APOE expression. Functional assays confirmed that PYCR1 promotes BCa cell proliferation and migration in an APOE‐dependent manner. GSEA analysis revealed that PYCR1 might promote tumour progression through the JAK2/STAT3 signalling pathway, which was subsequently confirmed by Western blot. Previous studies have documented that APOE facilitates thyroid carcinoma progression through the JAK2/STAT3 signalling pathway.^26^, ^44^, ^45^ On this basis, we hypothesised that PYCR1 promotes BCa progression by modulating the APOE/JAK2/STAT3 axis; this hypothesis was validated by rescue experiments and Western blot.

Endogenous and exogenous Co‐IP assays, together with IF analysis, confirmed the interaction between PYCR1 and NUDT5. Western blot and qPCR showed that NUDT5 knockdown did not alter PYCR1 expression at either the mRNA or protein level but significantly reduced APOE mRNA and protein levels. Rescue experiments further established NUDT5 as a critical mediator of PYCR1‐dependent APOE regulation. As an ADP‐ribose pyrophosphatase, NUDT5 can catalyse the synthesis of nuclear ATP from ADP‐ribose in the presence of pyrophosphate, thereby influencing chromatin remodelling and transcriptional regulation. Our findings indicate that NUDT5‐derived nuclear ATP promotes SP1‐mediated transcriptional activation of APOE, whereas oligomycin suppresses this effect, providing new mechanistic insights into the regulation of APOE by NUDT5. Nevertheless, this study has several limitations. Future studies using purified recombinant proteins, such as GST pull‐down assays or other biophysical binding approaches, are required to determine whether PYCR1 binds directly to NUDT5. In addition, further investigation is needed to fully elucidate the precise mechanisms by which nuclear ATP regulates transcription.

Platinum‐based chemotherapy constitutes the first‐line adjuvant therapy for BCa, yet its clinical utility is constrained by systemic toxicities.^46^, ^47^ Although immune checkpoint inhibitors have exhibited promising antitumor efficacy, these agents remain hindered by suboptimal patient response rates.^48^ Consequently, the development of innovative therapeutic strategies represents an urgent imperative.^49^ Using cell lines, organoid models, and animal models, the present study demonstrated that TH5427 effectively suppresses BCa progression and metastasis. Although TH5427 shows considerable promise as a therapeutic strategy targeting NUDT5 enzymatic activity, several challenges must be addressed before its clinical translation. Future preclinical and clinical studies should comprehensively evaluate its potential toxicity, pharmacokinetic properties, and long‐term safety. Moreover, although TH5427 exhibits selectivity toward NUDT5, potential off‐target effects cannot be completely excluded. Future studies should therefore explore optimised dosing regimens and rational combination strategies, such as combining TH5427 with immunotherapy or antibody–drug conjugate therapy, to enhance therapeutic efficacy while minimising adverse effects.

To our knowledge, the present study is the first to systematically demonstrate that PYCR1 promotes BCa progression by interacting with NUDT5 and regulating the APOE/JAK2/STAT3 signalling axis. These findings uncover a non‐classical oncogenic role of PYCR1 in BCa and delineate a previously unrecognised molecular mechanism by which it drives BCa proliferation and metastasis. Moreover, the NUDT5‐targeted small molecule inhibitor TH5427 exhibits potent inhibitory effects on BCa progression, suggesting its potential as a promising therapeutic candidate for clinical management.

## 5 CONCLUSION

In summary, our study identifies a previously unrecognised PYCR1/NUDT5/APOEJAK2/STAT3 regulatory axis. PYCR1 interacts with NUDT5 and promotes NUDT5‐mediated nuclear ATP generation, thereby sustaining SP1‐driven APOE transcription and subsequently activating JAK2/STAT3 signalling. Functional rescue experiments further support the critical roles of NUDT5 catalytic activity and APOE in mediating the oncogenic effects of PYCR1. Moreover, pharmacological inhibition of NUDT5 with TH5427 suppresses BCa progression both in vitro and in vivo. Collectively, these findings expand our understanding of the noncanonical oncogenic functions of PYCR1 and highlight NUDT5 as a potential therapeutic target in BCa.

## AUTHOR CONTRIBUTIONS

Xudong Yao, Shiyu Mao, Bing Shen and Xiaojun Zhu conceived the study. Haotian Chen, Shuya Ji and Pengfei Wu drafted the manuscript. Haotian Chen, Ji Liu and Wentao Zhang prepared the figures and tables. Junfeng Zhang and Jingcheng Zhang interpreted the data. Zhijing Zhang and YanLing Liu polished the article. Yongqiang Liu and Chengqi Jin collected the references. Sichen Di and Yang Wu participated in the discussion and gave constructive comments. Yadong Guo contributed to the revision of the manuscript. All authors have read and agreed to the published version of the manuscript.

## CONFLICT OF INTEREST STATEMENT

The authors declare no conflict of interest.

## ETHICS STATEMENT

This study was approved by the Ethics Committee of STPH (No. 2021KN108). All procedures were performed in accordance with the ethical principles set forth in the Declaration of Helsinki. Data confidentiality and participant privacy were rigorously safeguarded throughout the study. All animal experiments conducted in this study were approved by the Ethics Review Committee of Shanghai Tenth People's Hospital.

## Supporting information



Supporting Information

Supporting Information

Supporting Information

Supporting Information

Supporting Information

Supporting Information

Supporting Information

Supporting Information

Supporting Information

Supporting Information

Supporting Information

Supporting Information

Supporting Information

Supporting Information

## Data Availability

The data and materials in the current study are available from the corresponding author on reasonable request.

## References

[1] Siegel RL , Kratzer TB , Wagle NS , Sung H , Jemal A . Cancer statistics, 2026. CA Cancer J Clin. 2026;76(1):e70043. 10.3322/caac.70043 PMC12798275 41528114

[2] Galsky MD , Ballas L , Kamat AM . Muscle‐invasive bladder cancer: a watershed moment. J Clin Oncol. 2025;43(27):3041‐3051. doi:10.1200/JCO‐25‐00718 10.1200/JCO-25-00718 40763316

[3] Lin J , Rao D , Zhang M , Gao Q . Metabolic reprogramming in the tumor microenvironment of liver cancer. J Hematol Oncol. 2024;17(1):6. doi:10.1186/s13045‐024‐01527‐8 10.1186/s13045-024-01527-8 PMC10832230 38297372

[4] Tharp KM , Kersten K , Maller O , et al. Tumor‐associated macrophages restrict CD8+ T cell function through collagen deposition and metabolic reprogramming of the breast cancer microenvironment. Nat Cancer. 2024;5(7):1045‐1062. doi:10.1038/s43018‐024‐00775‐4 10.1038/s43018-024-00775-4 PMC12204312 38831058

[5] Fan G , Yu Bo , Tang L , et al. TSPAN8^+^ myofibroblastic cancer–associated fibroblasts promote chemoresistance in patients with breast cancer. Sci Transl Med. 2024;16(741):eadj5705. doi:10.1126/scitranslmed.adj5705 10.1126/scitranslmed.adj5705 38569015

[6] Lawson H , Holt‐Martyn JP , Dembitz V , et al. The selective prolyl hydroxylase inhibitor IOX5 stabilizes HIF‐1α and compromises development and progression of acute myeloid leukemia. Nat Cancer. 2024;5(6):916‐937. doi:10.1038/s43018‐024‐00761‐w 10.1038/s43018-024-00761-w PMC11208159 38637657

[7] Kay EJ , Paterson K , Riera‐Domingo C , et al. Cancer‐associated fibroblasts require proline synthesis by PYCR1 for the deposition of pro‐tumorigenic extracellular matrix. Nat Metab. 2022;4(6):693‐710. doi:10.1038/s42255‐022‐00582‐0 10.1038/s42255-022-00582-0 PMC9236907 35760868

[8] Guo P , Wu C , Wang T , et al. The key enzyme PYCR1 in proline metabolism: a dual driver of cancer progression and fibrotic remodeling. J Enzyme Inhib Med Chem. 2025;40(1):2545620. doi:10.1080/14756366.2025.2545620 10.1080/14756366.2025.2545620 PMC12406333 40891362

[9] Liu S , Lao Y , Cheng L , et al. Integrating virtual screening and molecular dynamics simulations to identify emodin as a PYCR1 inhibitor modulating docetaxel sensitivity in prostate cancer. J Enzyme Inhib Med Chem. 2026;41(1):2622725. doi:10.1080/14756366.2026.2622725 10.1080/14756366.2026.2622725 PMC12893152 41665114

[10] Wang D , Wang L , Zhang Y , Yan Z , Liu L , Chen G . PYCR1 promotes the progression of non‐small‐cell lung cancer under the negative regulation of miR‐488. Biomed Pharmacother. 2019;111:588‐595. doi:10.1016/j.biopha.2018.12.089 10.1016/j.biopha.2018.12.089 30605882

[11] Xu Y , Zuo W , Wang X , et al. Deciphering the effects of PYCR1 on cell function and its associated mechanism in hepatocellular carcinoma. Int J Biol Sci. 2021;17(9):2223‐2239. doi:10.7150/ijbs.58026 10.7150/ijbs.58026 PMC8241733 34239351

[12] Cui B , He B , Huang Y , et al. Pyrroline‐5‐carboxylate reductase 1 reprograms proline metabolism to drive breast cancer stemness under psychological stress. Cell Death Dis. 2023;14(10):682. doi:10.1038/s41419‐023‐06200‐5 10.1038/s41419-023-06200-5 PMC10579265 37845207

[13] Li Z , Jiang Y , Liu J , et al. Exosomes from PYCR1 knockdown bone marrow mesenchymal stem inhibits aerobic glycolysis and the growth of bladder cancer cells via regulation of the EGFR/PI3K/AKT pathway. Int J Oncol. 2023;63(1):84. doi:10.3892/ijo.2023.5532 10.3892/ijo.2023.5532 PMC10552724 37293856

[14] Du S , Sui Y , Ren W , Zhou J , Du C . PYCR1 promotes bladder cancer by affecting the Akt/Wnt/β‐catenin signaling. J Bioenerg Biomembr. 2021;53(2):247‐258. doi:10.1007/s10863‐021‐09887‐3 10.1007/s10863-021-09887-3 33689096

[15] Xiao S , Chen J , Wei Y , Song W . BHLHE41 inhibits bladder cancer progression via regulation of PYCR1 stability and thus inactivating PI3K/AKT signaling pathway. Eur J Med Res. 2024;29(1):302. doi:10.1186/s40001‐024‐01889‐2 10.1186/s40001-024-01889-2 PMC11134742 38811952

[16] Li Z , Jiang Q , Yang Q , Zhou Y , Wang J . Hypoxia‐induced PYCR1 regulates glycolysis and histone lactylation to promote bladder cancer progression and metastasis via SLC6A14/Glutamine metabolism. Cancer Biol Ther. 2025;26(1):2546219. doi:10.1080/15384047.2025.2546219 10.1080/15384047.2025.2546219 PMC12355718 40808274

[17] Maillard M , Nishii R , Vu HS , et al. The NUDIX hydrolase NUDT5 regulates thiopurine metabolism and cytotoxicity. J Clin Invest. 2025;135(14):e190443. doi:10.1172/JCI190443 10.1172/JCI190443 PMC12259257 40662362

[18] Qi H , Grace Wright RH , Beato M , Price BD . The ADP‐ribose hydrolase NUDT5 is important for DNA repair. Cell Rep. 2022;41(12):111866. doi:10.1016/j.celrep.2022.111866 10.1016/j.celrep.2022.111866 36543120

[19] Wright RHG , Lioutas A , Le Dily F , et al. ADP‐ribose–derived nuclear ATP synthesis by NUDIX5 is required for chromatin remodeling. Science. 2016;352(6290):1221‐1225. doi:10.1126/science.aad9335 10.1126/science.aad9335 27257257

[20] Ding H , Wu C , Sun W , et al. NUDT5 determines the fate of head and neck squamous cell carcinoma cells under endoplasmic reticulum stress by catalyzing nuclear ATP production to promote DNA repair. Oral Oncol. 2023;141:106397. doi:10.1016/j.oraloncology.2023.106397 10.1016/j.oraloncology.2023.106397 37156197

[21] Sun R , A J , Yu H , et al. Proteomic landscape profiling of primary prostate cancer reveals a 16‐protein panel for prognosis prediction. Cell Rep Med. 2024;5(8):101679. doi:10.1016/j.xcrm.2024.101679 10.1016/j.xcrm.2024.101679 PMC11384950 39168102

[22] Qian J , Ma Y , Tahaney WM , et al. The novel phosphatase NUDT5 is a critical regulator of triple‐negative breast cancer growth. Breast Cancer Res. 2024;26(1):23. doi:10.1186/s13058‐024‐01778‐w 10.1186/s13058-024-01778-w PMC10845800 38317231

[23] Page BDG , Valerie NCK , Wright RHG , et al. Targeted NUDT5 inhibitors block hormone signaling in breast cancer cells. Nat Commun. 2018;9(1):250. doi:10.1038/s41467‐017‐02293‐7 10.1038/s41467-017-02293-7 PMC5772648 29343827

[24] Sukalskaia A , Karner A , Pugnetti A , Weber F , Plochberger B , Dutzler R . Interactions between TTYH2 and APOE facilitate endosomal lipid transfer. Nature. 2025;644(8075):273‐279. doi:10.1038/s41586‐025‐09200‐x 10.1038/s41586-025-09200-x PMC12328215 40562935

[25] Jackson RJ , Hyman BT , Serrano‐Pozo A . Multifaceted roles of APOE in Alzheimer disease. Nat Rev Neurol. 2024;20(8):457‐474. doi:10.1038/s41582‐024‐00988‐2 10.1038/s41582-024-00988-2 PMC12185264 38906999

[26] Huang J , Sun W , Wang Z , et al. FTO suppresses glycolysis and growth of papillary thyroid cancer via decreasing stability of APOE mRNA in an N6‐methyladenosine‐dependent manner. J Exp Clin Cancer Res. 2022;41(1):42. doi:10.1186/s13046‐022‐02254‐z 10.1186/s13046-022-02254-z PMC8796435 35090515

[27] Furuya H , Sakatani T , Tanaka S , et al. Bladder cancer risk stratification with the Oncuria 10‐plex bead‐based urinalysis assay using three different Luminex xMAP instrumentation platforms. J Transl Med. 2024;22(1):8. doi:10.1186/s12967‐023‐04811‐2 10.1186/s12967-023-04811-2 PMC10763405 38167321

[28] Zheng Z , Mao S , Zhang W , et al. Dysregulation of the immune microenvironment contributes to malignant progression and has prognostic value in bladder cancer. Front Oncol. 2020;10:542492. doi:10.3389/fonc.2020.542492 10.3389/fonc.2020.542492 PMC7773013 33392066

[29] Shen L , Zhang J , Zheng Z , et al. PHGDH inhibits ferroptosis and promotes malignant progression by upregulating SLC7A11 in bladder cancer. Int J Biol Sci. 2022;18(14):5459‐5474. doi:10.7150/ijbs.74546 10.7150/ijbs.74546 PMC9461664 36147463

[30] Hsu En‐C , Rice MA , Bermudez A , et al. Trop2 is a driver of metastatic prostate cancer with neuroendocrine phenotype via PARP1. Proc Natl Acad Sci U S A. 2020;117(4):2032‐2042. doi:10.1073/pnas.1905384117 10.1073/pnas.1905384117 PMC6994991 31932422

[31] Liu X , Chen Z , Xu C , et al. Repression of hypoxia‐inducible factor α signaling by Set7‐mediated methylation. Nucleic Acids Res. 2015;43(10):5081‐5098. doi:10.1093/nar/gkv379 10.1093/nar/gkv379 PMC4446437 25897119

[32] Chang DJ , Paik YK , Leren TP , Walker DW , Howlett GJ , Taylor JM . Characterization of a human apolipoprotein E gene enhancer element and its associated protein factors. J Biol Chem. 1990;265(16):9496‐9504. doi:10.1016/S0021‐9258(19)38877‐5 2188976

[33] Gafencu AV , Robciuc MR , Fuior E , Zannis VI , Kardassis D , Simionescu M . Inflammatory signaling pathways regulating ApoE gene expression in macrophages. J Biol Chem. 2007;282(30):21776‐21785. doi:10.1074/jbc.M611422200 10.1074/jbc.M611422200 17553793

[34] Liu S , Zhang X , Wang W , et al. Metabolic reprogramming and therapeutic resistance in primary and metastatic breast cancer. Mol Cancer. 2024;23(1):261. doi:10.1186/s12943‐024‐02165‐x 10.1186/s12943-024-02165-x PMC11580516 39574178

[35] Zheng Ke , Sha N , Hou G , et al. IGF1R‐phosphorylated PYCR1 facilitates ELK4 transcriptional activity and sustains tumor growth under hypoxia. Nat Commun. 2023;14(1):6117. doi:10.1038/s41467‐023‐41658‐z 10.1038/s41467-023-41658-z PMC10542766 37777542

[36] Zhang L , Zhao X , Wang E , et al. PYCR1 promotes the malignant progression of lung cancer through the JAK‐STAT3 signaling pathway via PRODH‐dependent glutamine synthesize. Transl Oncol. 2023;32:101667. doi:10.1016/j.tranon.2023.101667 10.1016/j.tranon.2023.101667 PMC10106966 37018868

[37] Shin JH , Kim JY , Kim M‐J , et al. PYCR1 drives lung cancer progression through functional interactions with EGFR and TLR signaling pathways. Exp Mol Med. 2025;57(11):2559‐2573. doi:10.1038/s12276‐025‐01577‐z 10.1038/s12276-025-01577-z PMC12686397 41254241

[38] Wang H , Xu Mu , Zhang T , et al. PYCR1 promotes liver cancer cell growth and metastasis by regulating IRS1 expression through lactylation modification. Clin Transl Med. 2024;14(10):e70045. doi:10.1002/ctm2.70045 10.1002/ctm2.70045 PMC11488319 39422696

[39] Ding X , Zhang E , Huang Z , et al. Glutamine metabolism reprogramming promotes bladder cancer progression via PYCR1: a multi‐omics and functional validation study. J Transl Med. 2025;23(1):1277. doi:10.1186/s12967‐025‐07386‐2 10.1186/s12967-025-07386-2 PMC12613347 41233804

[40] Stum MG , Tadenev ALD , Seburn KL , et al. Genetic analysis of Pycr1 and Pycr2 in mice. Genetics. 2021;218(1):iyab048. doi:10.1093/genetics/iyab048 10.1093/genetics/iyab048 PMC8128379 33734376

[41] Luo Y , Yu J , Lin Z , et al. Metabolic characterization of sphere‐derived prostate cancer stem cells reveals aberrant urea cycle in stemness maintenance. Int J Cancer. 2024;155(4):742‐755. doi:10.1002/ijc.34967 10.1002/ijc.34967 38647131

[42] Lou X , Shi Y , Zhao F , et al. Pancreatic neuroendocrine tumors secrete Apolipoprotein E to induce tip endothelial cells that remodel the tumor–stroma ratio and promote cancer progression. Cancer Res. 2025;85(15):2805‐2819. doi:10.1158/0008‐5472.CAN‐24‐2528 10.1158/0008-5472.CAN-24-2528 PMC12314519 40623046

[43] Wang Q , Wu Y , Long Y , et al. AR+TREM2+ macrophage induced pathogenic immunosuppression promotes prostate cancer progression. Nat Commun. 2025;16(1):6964. doi:10.1038/s41467‐025‐62381‐x 10.1038/s41467-025-62381-x PMC12307935 40730555

[44] Fu X , Sun Z , Long Q , et al. Glycosides from Buyang Huanwu Decoction inhibit atherosclerotic inflammation via JAK/STAT signaling pathway. Phytomedicine. 2022;105:154385. doi:10.1016/j.phymed.2022.154385 10.1016/j.phymed.2022.154385 35987015

[45] Xiang Lu , Wang Y , Liu Si , et al. Quercetin attenuates KLF4‐mediated phenotypic switch of VSMCs to macrophage‐like cells in atherosclerosis: a critical role for the JAK2/STAT3 pathway. Int J Mol Sci. 2024;25(14):7755. doi:10.3390/ijms25147755 10.3390/ijms25147755 PMC11277168 39062998

[46] Mertens LS , van der Heijden MS . Accelerating neoadjuvant chemotherapy for muscle‐invasive bladder cancer. Lancet Oncol. 2024;25(2):156‐157. doi:10.1016/S1470‐2045(23)00626‐5 10.1016/S1470-2045(23)00626-5 38142700

[47] Tian X , Jia H , Liu Y , et al. Clinicopathological features and prognosis of small cell carcinoma of the urinary bladder. Asian J Urol. 2025;12(3):393‐401. doi:10.1016/j.ajur.2024.10.008 10.1016/j.ajur.2024.10.008 PMC12490661 41049815

[48] Boll LM , Vázquez Montes de Oca S , Camarena ME , et al. Predicting immunotherapy response of advanced bladder cancer through a meta‐analysis of six independent cohorts. Nat Commun. 2025;16(1):1213. doi:10.1038/s41467‐025‐56462‐0 10.1038/s41467-025-56462-0 PMC11842772 39979258

[49] Liu S , Liu Z , Piao C , et al. Flavokawain A is a natural inhibitor of PRMT5 in bladder cancer. J Exp Clin Cancer Res. 2022;41(1):293. doi:10.1186/s13046‐022‐02500‐4 10.1186/s13046-022-02500-4 PMC9533510 36199122

