# Supplementary material for: Pyrroline‐5‐carboxylate reductase 1 interacts with Nudix hydrolase 5 to drive bladder cancer malignant progression via activation of the APOE/JAK2/STAT3 signalling axis
Source: Clin Transl Med. 2026 Sep 18;16(9):e70840. doi: 10.1002/ctm2.70840 (PMC13588273; doi:10.1002/ctm2.70840)
Supplement: Supplementary file 1 [file CTM2-16-e70840-s007.docx]

**Supplementary Table 4. Clinicopathological characteristics of patients included in the bladder cancer tissue microarray**

| **Number** | **Gender** | **Age** | **T stage** | **Grade** | **OS time（month）** | **OS** |
| --- | --- | --- | --- | --- | --- | --- |
| 1 | male | 62 | T1 | low | 78 | 0 |
| 2 | male | 58 | T1 | low | 59 | 0 |
| 3 | female | 65 | T1 | low | 119 | 0 |
| 4 | male | 75 | T1 | low | 54 | 0 |
| 5 | male | 61 | T1 | low | 50 | 0 |
| 6 | male | 71 | T1 | low | 58 | 1 |
| 7 | male | 78 | T1 | low | 62 | 0 |
| 8 | male | 54 | T1 | low | 83 | 0 |
| 9 | male | 63 | T1 | low | 90 | 0 |
| 10 | female | 59 | T1 | low | 83 | 1 |
| 11 | male | 60 | T2 | high | 40 | 0 |
| 12 | male | 62 | T1 | low | 48 | 0 |
| 13 | male | 57 | T1 | low | 50 | 0 |
| 14 | male | 62 | T1 | low | 39 | 0 |
| 15 | male | 73 | T1 | low | 32 | 0 |
| 16 | male | 69 | T1 | low | 30 | 0 |
| 17 | male | 55 | T1 | low | 62 | 1 |
| 18 | male | 61 | T2 | high | 70 | 0 |
| 19 | male | 65 | Ta | low | 90 | 1 |
| 20 | female | 59 | Ta | low | 88 | 1 |
| 21 | male | 76 | Ta | low | 110 | 0 |
| 22 | male | 63 | Ta | low | 102 | 0 |
| 23 | male | 80 | Ta | low | 61 | 1 |
| 24 | male | 52 | Ta | low | 80 | 0 |
| 25 | male | 60 | Ta | low | 40 | 0 |
| 26 | male | 72 | Ta | low | 35 | 0 |
| 27 | male | 74 | T2 | high | 50 | 0 |
| 28 | male | 64 | Ta | low | 78 | 0 |
| 29 | male | 77 | T2 | high | 74 | 0 |
| 30 | male | 58 | Ta | low | 68 | 0 |
| 31 | female | 56 | Ta | low | 62 | 0 |
| 32 | male | 62 | Ta | low | 94 | 0 |
| 33 | male | 61 | T2 | high | 90 | 1 |
| 34 | male | 63 | T2 | high | 110 | 0 |
| 35 | male | 49 | T2 | high | 107 | 0 |
| 36 | male | 68 | T3 | high | 75 | 1 |
| 37 | male | 75 | T1 | low | 70 | 0 |
| 38 | female | 79 | T2 | high | 84 | 0 |
| 39 | male | 60 | T2 | high | 88 | 0 |
| 40 | male | 65 | T2 | high | 40 | 1 |
| 41 | male | 57 | T2 | high | 37 | 1 |
| 42 | male | 64 | T2 | high | 65 | 1 |
| 43 | male | 59 | T3 | high | 52 | 0 |
| 44 | male | 53 | T2 | high | 42 | 0 |
| 45 | male | 62 | T2 | high | 70 | 0 |
| 46 | female | 61 | T2 | high | 72 | 1 |
| 47 | male | 54 | T2 | high | 100 | 0 |
| 48 | male | 70 | T2 | high | 92 | 0 |
| 49 | male | 73 | T2 | high | 77 | 0 |
| 50 | male | 76 | T2 | high | 62 | 0 |
| 51 | female | 63 | T2 | high | 66 | 1 |
| 52 | male | 74 | T2 | high | 82 | 0 |
| 53 | male | 55 | T1 | low | 64 | 0 |
| 54 | male | 60 | T3 | high | 74 | 0 |
| 55 | female | 78 | T3 | high | 84 | 0 |
| 56 | male | 67 | T2 | high | 45 | 0 |
| 57 | male | 63 | T3 | high | 46 | 0 |
| 58 | male | 73 | Ta | low | 48 | 0 |
| 59 | male | 75 | T4 | high | 57 | 1 |
| 60 | male | 51 | T3 | high | 40 | 0 |
| 61 | male | 65 | T3 | high | 56 | 1 |
| 62 | male | 56 | T2 | high | 70 | 0 |
| 63 | male | 62 | T3 | high | 52 | 1 |
| 64 | male | 59 | T1 | low | 88 | 0 |
| 65 | male | 61 | T3 | high | 40 | 0 |
| 66 | male | 80 | T3 | high | 50 | 0 |
| 67 | male | 71 | T3 | high | 68 | 1 |
| 68 | male | 77 | T4 | high | 69 | 0 |
| 69 | female | 63 | T3 | high | 70 | 1 |
| 70 | male | 69 | T3 | high | 50 | 1 |
| 71 | male | 74 | T4 | high | 42 | 0 |
| 72 | male | 47 | T4 | high | 41 | 1 |
| 73 | male | 76 | T4 | high | 36 | 0 |
| 74 | male | 73 | T1 | low | 48 | 0 |
| 75 | male | 78 | T1 | low | 78 | 1 |
| 76 | male | 66 | T3 | high | 32 | 0 |
| 77 | male | 58 | T4 | high | 38 | 0 |
| 78 | male | 75 | T4 | high | 42 | 1 |
| 79 | female | 73 | T4 | high | 55 | 1 |
| 80 | male | 79 | T3 | high | 64 | 1 |
| 81 | male | 64 | T4 | high | 50 | 0 |
| 82 | male | 70 | T3 | high | 60 | 1 |
| 83 | male | 77 | T4 | high | 18 | 1 |
| 84 | male | 74 | T4 | high | 35 | 0 |
| 85 | male | 68 | T3 | high | 30 | 0 |
| 86 | male | 76 | T4 | high | 25 | 1 |
