# Supplementary material for: Pyrroline‐5‐carboxylate reductase 1 interacts with Nudix hydrolase 5 to drive bladder cancer malignant progression via activation of the APOE/JAK2/STAT3 signalling axis
Source: Clin Transl Med. 2026 Sep 18;16(9):e70840. doi: 10.1002/ctm2.70840 (PMC13588273; doi:10.1002/ctm2.70840)
Supplement: Supplementary file 2 [file CTM2-16-e70840-s008.docx]

**PYCR1 interacts with NUDT5 to drive bladder cancer malignant progression via activation of the APOE/JAK2/STAT3 signaling axis**

Haotian Chen^1,2,3 †^, Shuya Ji^4, †^, Pengfei Wu^1,2 †^, Ji Liu^1,2,3 †^, Wentao Zhang^1,2, †^, Yadong Guo^1,2^, Jingcheng Zhang^1,2,3^, Zhijing Zhang^1,2,3^, YanLing Liu^5^, Yongqiang Liu^1,2,3^, Chengqi Jin^1,2,3^, Sichen, Di^1,2,3^, Yang Wu^1,2,3^, Junfeng Zhang^1,2 *^, Xiaojun Zhu^6 *^, Shiyu Mao^1,2 *^, Bing Shen^1,2 *^, Xudong Yao^1,2 *^

1.Department of Urology, Shanghai Tenth People’s Hospital, Tongji University School of Medicine, Shanghai, China

2.Urologic Cancer Institute, Tongji University School of Medicine, Shanghai, China

3.School of Medicine, Tongji University, Shanghai, China

4.Department of Oncology, Gongli Hospital of Shanghai Pudong New Area，Shanghai，China

5.State Key Laboratory of Pollution Control and Resources Reuse, Tongji Advanced Membrane Technology Center, Tongji University, Shanghai 200092, China.

6.Department of Urology Surgery, The Affiliated Hospital of Inner Mongolia Medical University, Hohhot, China.

† Authors contributed equally

These authors contributed equally to this work

*Corresponding author:

Prof. Xudong Yao

Department of Urology, Shanghai Tenth People’s Hospital, Tongji University, Shanghai 200072, P. R. China Email: [yaoxudong1967@163.com](mailto:yaoxudong1967@163.com)

Dr. Shiyu Mao

Department of Urology, Shanghai Tenth People’s Hospital, Tongji University, Shanghai 200072, China. Email: [maoshiyu1144@sina.com](mailto:maoshiyu1144@sina.com)

Prof. Xiaojun Zhu

Department of Urology Surgery, The Affiliated Hospital of Inner Mongolia Medical University, Hohhot, China. Email: [2728600785@qq.com](mailto:2728600785@qq.com)

Prof. Bing Shen

Department of Urology, Shanghai Tenth People’s Hospital, Tongji University, Shanghai 200072, P. R. China Email: [urodrshenbing@shsmu.edu.cn](mailto:urodrshenbing@shsmu.edu.cn)

Dr. Junfeng Zhang

Department of Urology, Shanghai Tenth People’s Hospital, Tongji University, Shanghai 200072, China. Email: [zjfdipan@126.com](mailto:zjfdipan@126.com)

**Supplementary Methods**

**Cell culture and cell lines**

All cell lines applied in the present work were sourced from the Chinese Academy of Sciences (Shanghai, China), and cell identity was verified via short tandem repeat (STR) profiling before all experimental procedures were initiated. The human BCa cell lines T24, UMUC3, RT4 and 5637, together with the immortalized human normal bladder epithelial cell line SV-HUC-1, were employed. Specifically, RT4 cells were maintained in McCoy's 5A medium (Thermo Fisher Scientific Inc., USA). SV-HUC-1 cells were cultured with F12K medium (Sigma-Aldrich, USA), while T24, UMUC3 and 5637 cells were grown in RPMI-1640 medium (Thermo, USA). All culture media were added with 10% fetal calf serum (FCS; Thermo, USA), and cells were incubated in a humidified incubator at 37°C with 5% CO₂.

**Animal experiments**

For subcutaneous xenograft assays, BCa cells were resuspended in phosphate-buffered saline (PBS) at a density of 1×10⁶ cells/mL. A total volume of 100 µL cell suspension was inoculated into the right axillary fossa of nude mice. After 4 weeks, the mice were euthanized, and the subcutaneous tumors were harvested for subsequent experiments. Tumor volume was calculated using the formula: V =π/6 × length × width². For lung metastasis assays, luciferase transduced BCa cells (1×10⁶ cells) were administered via intravenous injection. Mouse body weight was measured at regular intervals, and bioluminescence imaging analysis was conducted to assess the degree of pulmonary tumor metastasis.

One week after successful establishment of the subcutaneous xenograft tumors, mice received intraperitoneal injections of TH5427 (50 mg/kg) or vehicle (DMSO). TH5427 or vehicle was administered for 5 consecutive days followed by a 2-day treatment-free interval each week, for a total treatment duration of 3 weeks. Tumor volumes were measured weekly. At 4 weeks after tumor cell inoculation, the mice were sacrificed, and the tumors were excised and weighed.

**RNA isolation and Real Time- quantitative PCR**

Total RNA was extracted from BCa cells using a total RNA isolation kit (RK30129, Abclonal), and complementary DNA (cDNA) was synthesized with PrimeScript RT reagents (R323-01, Vazyme). Quantitative real-time polymerase chain reaction (RT-qPCR) was conducted with qPCR SYBR Green Master Mix (11201ES03, Yeasen). Target RNA relative expression was quantified using the 2^⁻△△Ct^ algorithm, with glyceraldehyde-3-phosphate dehydrogenase (GAPDH) serving as the internal housekeeping reference gene. The primers used in this study are included in Supplementary Table 1.

**RNA-sequencing**

Total RNA was extracted from BCa cells using Trizol reagent, followed by the construction of strand-specific libraries based on the dUTP method. Libraries were sequenced on an Illumina platform with paired-end 150 bp (PE150) reads. Raw reads were aligned to the human GRCh38 reference genome using HISAT2, and transcript assembly was conducted with StringTie. Differential expression analysis was performed using the edgeR package, with fragments per kilobase of transcript per million mapped reads (FPKM)-normalized counts. Genes with |fold change| > 1.2 and P-value < 0.05 were defined as differentially expressed genes (DEGs).

**Western blot and Co-IP**

Total protein was isolated via RIPA lysis buffer (HATF00010, Yesean). Protein samples were fractionated through 10% sodium dodecyl sulfate-polyacrylamide gel electrophoresis (SDS-PAGE), followed by transfer onto nitrocellulose (NC) membranes (HATF00010,Millipore). The membrane was blocked using 5% skimmed milk powder for 1 hour, and then incubated with primary antibodies overnight at 4 °C. After three rinses in Tris-buffered saline containing Tween-20 (TBST), the membrane was incubated with secondary antibodies for 1 h at room temperature under gentle shaking. Protein bands were visualized using an enhanced chemiluminescence (ECL) substrate ( RM00021, Abclonal) on a TANON imaging system (Model No. 5200, Shanghai, China). Protein molecular weights were estimated by comparison with a prestained protein molecular weight marker electrophoresed in parallel on the same gel. The molecular weight values shown beside the blots indicate the approximate migration positions of the target proteins relative to the marker. Information on all antibodies used in this study has been provided in Supplementary Table 2.

Co-immunoprecipitation (Co-IP) assays were performed as follows: cells were lysed on ice for 30 minutes using lysis buffer (20118ES6, Yeasen) supplemented with a protease inhibitor cocktail (ST506, Beyotime). Whole-cell lysates were incubated with the indicated primary antibody overnight at 4°C, followed by incubation with Protein A/G agarose beads (36403ES03, Yeasen) for 3 hours at the same temperature. After four washes with lysis buffer, immunoprecipitated proteins and 10% of the input control were subjected to Western blot analysis. Conformation-specific horseradish peroxidase (HRP)-conjugated anti-mouse or anti-rabbit IgG secondary antibodies were utilized to selectively bind native IgG, thereby eliminating interference from IP antibody fragments. Detailed information of the antibodies used is provided in the supplementary materials.

**Immunohistochemistry (IHC) analysis**

For immunohistochemical (IHC) staining, BCa xenografts were deparaffinized and rehydrated via a graded alcohol series, followed by antigen retrieval in sodium citrate buffer. Tumor sections were blocked with PBS containing 3% bovine serum albumin (BSA), 0.25% Triton X-100, and 3% hydrogen peroxide (H₂O₂) for 1 hour at room temperature, then incubated with the specified primary antibodies overnight at 4°C. Following primary antibody incubation, sections were incubated with HRP-conjugated secondary antibodies, and immunoreactive signals were visualized using diaminobenzidine (DAB) as the chromogenic substrate. IHC results were independently evaluated by three pathologists. The proportion of positive cells was quantitatively analyzed using Image J software.

**Immunofluorescence (IF) and co-localization analysis**

BCa cells were fixed with 4% formaldehyde, permeabilized with 0.25% Triton X-100, and blocked with 3% BSA at room temperature for 1 hour. Cells were then incubated with the primary antibody, washed with TBST, and incubated with the corresponding secondary antibody. Stained samples were visualized using a high-resolution laser scanning confocal microscope (Carl Zeiss LSM 900, Jena, Germany). Co-localization was quantified via Pearson’s correlation coefficient (PCC) and line profile analysis. As previously described, PCC values were calculated using ImageJ from 50 cells, with data presented as mean ± 95% confidence interval (CI)(29, 30). For line profile analysis, a straight line was drawn across cells using the straight-line tool in ImageJ, and fluorescence intensity profiles along the line for both channels were extracted via the plot profile function. Tissue microarrays (TMAs) were constructed using 86 bladder cancer tissue specimens, which were subjected to IF staining for PYCR1 using a previously established protocol.

**Cell counting Kit-8 (CCK8) assay**

Cell proliferation was evaluated using the CCK-8 assay kit (K2268, Apexbio). Cells were seeded in 96-well plates at a density of 1×10³ cells per well. Over the subsequent 5 consecutive days, 100 μL of culture medium supplemented with 10 μL of CCK-8 reagent was added to each well daily. After incubation, the absorbance at 450 nm was detected using a microplate reader.

**EdU assay**

Cell proliferation was assessed using the EdU cell proliferation assay kit (K1075, Apexbio). Bladder cancer (BCa) cells were seeded in 96-well plates and cultured for 24 hours, followed by incubation with 10 μM EdU-containing medium for 2 hours. Cells were fixed with 4% paraformaldehyde for 15 minutes and permeabilized with 0.3% Triton X-100 for 15 minutes. Cells were then stained sequentially with Alexa Fluor Cy3 azide (30 minutes) and Hoechst Staining Solution (30 minutes), under dark conditions. Images were captured using a fluorescence microscope.

**Colony formation assay**

BCa cells were seeded in 6-well plates at a density of 500 cells per well, followed by culture in 2 mL of complete medium at 37°C with 5% CO₂ for 2 weeks. Proliferative colonies were fixed with 4% paraformaldehyde and stained with crystal violet solution. Visible colonies were subsequently imaged and counted manually.

**Migration and invasion assay**

Transwell chambers ( 84051ES12, Yesean) were employed for conventional cell migration and invasion assays. For the migration assay, 3×10⁴ cells suspended in 200 μL serum-free medium were seeded into the upper chambers, while 700 μL medium supplemented with 20% fetal bovine serum (FBS) was added to the lower compartments. Following 48 hours of incubation, migrated cells were fixed with 4% formaldehyde, stained with crystal violet, and quantified by counting five randomly selected fields per chamber. For the invasion assay, Matrigel-precoated Transwell chambers were used, and the experimental procedure was consistent with that of the migration assay.

**Scratch wound healing assay**

BCa cells were seeded in 6-well plates and cultured until reaching 90% confluence. A linear scratch was created using a 200 μL pipette tip, and cell debris was removed by washing with PBS. Cells were then incubated in serum-free medium, and scratch closure was monitored and imaged at predetermined time points.

**Proline content detection**

BCa cells were seeded in culture dishes and allowed to reach approximately 70%–80% confluence. The cells were then treated with PYCR1-IN-1 (10 μM) or an equivalent volume of DMSO for 24 h. After treatment, the cells were washed twice with ice-cold PBS, harvested by trypsinization, and counted using a viable cell-counting method. For each sample, 5 × 10⁶ cells were resuspended in 1 mL of extraction buffer and disrupted by sonication on ice. The cell lysates were heated in a boiling water bath for 10 min, cooled to room temperature, and centrifuged at 10000 × g for 10 min. The resulting supernatants were collected, and intracellular proline levels were measured using a Proline Content Assay Kit (BC0295; Solarbio, Beijing, China). Briefly, 0.25 mL of each supernatant was mixed with the corresponding detection reagents and heated in a water bath for 30 min according to the manufacturer’s instructions. After cooling, absorbance was measured at 520 nm, and proline concentrations were calculated using a standard curve. All experiments were performed with at least three independent biological replicates.

**Dual-luciferase reporter assay**

The dual-luciferase reporter assay was performed using a Dual-Luciferase Reporter Assay Kit (GM-040502A, Genomeditech). Briefly, reporter constructs containing the wild-type APOE promoter or an APOE promoter harboring mutations in the SP1-binding site were cloned into the pGL4 vector and transfected into HEK293T cells. At 48 h after transfection, the cells were harvested and lysed, and firefly and Renilla luciferase activities were measured according to the manufacturer’s instructions. Firefly luciferase activity was normalized to Renilla luciferase activity, and the resulting relative luciferase activity was subjected to statistical analysis.

**Nomogram development and validation**

Univariate and multivariate Cox regression analyses were performed to construct nomograms for predicting the prognostic impacts of target genes and other clinicopathological features in patients with BCa. The nomograms were validated by calibration plots and the concordance index (C-index) using the rms package (v5.1) in R ([https://cran.r-project.org/web/packages/rms/](https://cran.r-project.org/web/packages/rms/)).

**Supplementary figure legends**

**Supplementary figure 1. PYCR1 expression and its prognostic significance in multiple databases** (A-B) Volcano plots illustrate differentially expressed genes (DEGs) in the GSE13507 and Shanghai Tenth People Hospital (STPH) datasets. (C) Univariate Cox regression analysis of DEGs. (D, E) PYCR1 expression levels are elevated in distant metastases, high grade bladder cancer (BCa) based on the TCGA cohort. (F-I) PYCR1 is highly expressed in tumor tissues and correlated with advanced T stage, N stage, and high grade in the GSE13507 dataset. (J) Patients with high PYCR1 expression exhibit poorer prognosis in the GSE13507 dataset. (K) Univariate Cox regression analysis of PYCR1 and clinical characteristics. Data were presented as means ± SD, and *P* value was determined using Student's t-test. ^*^*P* < 0.05, ^**^*P* < 0.01, ^***^*P* < 0.001.

**Supplementary figure 2. PYCR1 facilitates the migration and epithelial–mesenchymal transition (EMT) of BCa cells.**

(A) qPCR was used to evaluate PYCR1 mRNA expression levels in normal urothelial and BCa cell lines. (B) qPCR was conducted to quantify mRNA expression levels upon PYCR1 knockdown. (C, D) Scratch healing assays assessed the influence of PYCR1 knockdown on BCa cell migration. (E) Western blot is performed to detect EMT-related proteins in BCa cells following PYCR1 knockdown. (F, G) A TCGA‑based nomogram is constructed and validated for prognostic reliability. (H) The ROC curve demonstrates the prognostic predictive ability of the model. Data were presented as means ± SD (n=3), and *P* value was determined using Student's t-test. ^*^*P* < 0.05, ^**^*P* < 0.01, ^***^*P* < 0.001.

**Supplementary figure 3. Construction of stable PYCR1 knockdown BCa cell lines.** (A-D) qPCR and Western blot analyses validate the efficiency of PYCR1 knockdown and overexpression in T24 and UMUC3 cell lines. Data were presented as means ± SD (n=3), and *P* value was determined using Student's t-test. ^*^*P* < 0.05, ^**^*P* < 0.01, ^***^*P* < 0.001.

**Supplementary figure 4. Generation of APOE knockdown BCa cell lines.** (A) qPCR analysis of SCARB1, APOA1, APOA2, APOC2, and APOE mRNA levels following PYCR1 knockdown. (B) Correlation analyses of PYCR1 mRNA expression with SCARB1, APOA1, APOA2, and APOC2 mRNA expression based on the TCGA dataset. (C-F) The efficiency of APOE knockdown in T24 and UMUC3 cell lines was confirmed by qPCR and Western blot analyses. Data were presented as means ± SD (n=3), and *P* value was determined using Student's t-test. ^*^*P* < 0.05, ^**^*P* < 0.01, ^***^*P* < 0.001.

**Supplementary figure 5. PYCR1 regulates APOE expression in a NUDT5-dependent manner.**

(A) Exogenous co-immunoprecipitation assays demonstrated the binding between Flag-tagged PYCR1 and HA-tagged NUDT5 in HEK293T cells. (B, C) The efficiency of NUDT5 knockdown or overexpression in T24 and UMUC3 cell lines was confirmed by qPCR and Western blot. (D, E) Western blot and qPCR analyses showed that NUDT5 overexpression rescued the reduction in APOE expression caused by PYCR1 knockdown. (F, G) The PYCR1 overexpression induced increase in APOE expression was reversed by NUDT5 knockdown, as confirmed by Western blot and qPCR analyses. (H, I) Western blot and PCR analyses showed that wild-type NUDT5 (NUDT5-WT) rescued the reduction in APOE expression caused by PYCR1 knockdown, whereas the catalytically inactive NUDT5 mutant (NUDT5-Mut) failed to do so. (J) Intracellular proline levels were measured in T24 cells following PYCR1-IN-1 treatment. (K) Western blot was performed to examine the protein levels of PYCR1, NUDT5, APOE, and JAK2/STAT3 signaling pathway following PYCR1-IN-1 treatment. (L, M) Colony formation and Transwell assays were performed to evaluate the enzymatic activity-independent effects of PYCR1 on cell proliferation and migration. The corresponding quantitative results are presented as bar graphs. Scale bar=200um. (N) Multiplex immunofluorescence (mIF) staining of PYCR1, NUDT5, and APOE in BCa tissues. Data were presented as means ± SD (n=3), and *P* value was determined using Student's t-test. ^*^*P* < 0.05, ^**^*P* < 0.01, ^***^*P* < 0.001.

**Supplementary figure 6. NUDT5-dependent ATP production sustains SP1-mediated APOE transcription.**

(A) Potential transcription factors regulating APOE were screened using the JASPAR, TFBS, GTRD, and ChIP-Atlas databases. (B) Analysis of public ChIP-seq datasets revealed SP1-binding peaks within the APOE promoter region. (C, D) qPCR and Western blot were performed to assess APOE mRNA and protein levels following SP1 knockdown. (E) Potential SP1-binding sites within the APOE promoter were predicted using the JASPAR database. (F) Construction of reporter vectors containing the wild-type APOE (APOE-WT) promoter or an APOE promoter harboring a mutated SP1-binding site (APOE-Mut). (G) Dual-luciferase reporter assay was performed to evaluate the effects of SP1 on the transcriptional activities of the APOE-WT and APOE-Mut promoter constructs in the presence or absence of oligomycin. (H) Nuclear ATP levels were measured in T24 cells following oligomycin treatment. (I) Western blot analysis was performed to assess SP1 and APOE protein levels and changes in JAK2/STAT3 signaling following oligomycin treatment. (J, K) Colony formation and Transwell assays were performed to evaluate whether APOE overexpression rescued the impaired proliferation and migration caused by NUDT5 knockdown. (L) Western blot was performed to determine whether APOE overexpression rescued the suppression of JAK2/STAT3 signaling induced by NUDT5 knockdown. (M) Wound healing assays showed that NUDT5 overexpression rescued the impaired cell migration caused by PYCR1 knockdown. Scale bar=200um. (N) Wound healing assays showed that NUDT5 knockdown reversed the increase in cell migration induced by PYCR1 overexpression. Scale bar=200um.Data were presented as means ± SD (n=3), and *P* value was determined using Student's t-test. ^*^*P* < 0.05, ^**^*P* < 0.01, ^***^*P* < 0.001.

**Supplementary figure 7. TH5427 suppresses the proliferation and migration of BCa cells.**

(A) The CCK‑8 assay determines the IC50 value of TH5427 in UMUC3 cells. (B, C) Colony formation assays verify the effect of NUDT5 knockdown and TH5427 treatment on UMUC3 cell proliferation. (D, E) Transwell assays evaluate the effects of NUDT5 knockdown and TH5427 treatment on UMUC3 cell migration. Scale bar=200um. (F) ATP assay measurements intracellular ATP levels in BCa cells following NUDT5 knockdown and TH5427 treatment. (G) Cells were treated with TH5427 (0, 20, 40, 80 μM) for 48 h, and NUDT5 expression was analyzed by Western blot. (H) Western blot analysis was performed to assess the effects of TH5427 treatment on NUDT5 and APOE protein levels and JAK2/STAT3 signaling. (I) H&E staining of mouse heart, liver, spleen, lung, and kidney tissues was performed to evaluate the biosafety and potential toxicity of TH5427. Data were presented as means ± SD (n=3), and *P* value was determined using Student's t-test. ^*^*P* < 0.05, ^**^*P* < 0.01, ^***^*P* < 0.001.
