# Supplementary material for: Pyrroline‐5‐carboxylate reductase 1 interacts with Nudix hydrolase 5 to drive bladder cancer malignant progression via activation of the APOE/JAK2/STAT3 signalling axis
Source: Clin Transl Med. 2026 Sep 18;16(9):e70840. doi: 10.1002/ctm2.70840 (PMC13588273; doi:10.1002/ctm2.70840)
Supplement: Supplementary file 3 [file CTM2-16-e70840-s012.docx]

**NUDT5-WT**

>NP_054861.2 ADP-sugar pyrophosphatase isoform 1 [Homo sapiens]

1	MESQEPTESS QNGKQYIISE ELISEGKWVK LEKTTYMDPT GKTRTWESVK

51	RTTRKEQTAD GVAVIPVLQR TLHYECIVLV KQFRPPMGGY CIEFPAGLID

101	DGETPEAAAL RELEEETGYK GDIAECSPAV CMDPGLSNCT IHIVTVTING

151	DDAENARPKP KPGDGEFVEV ISLPKNDLLQ RLDALVAEEH LTVDARVYSY

201	ALALKHANAK PFEVPFLKF

**NUDT5-Mut**

>NP_054861.2 ADP-sugar pyrophosphatase isoform 1 [Homo sapiens]

1	MESQEPTESS QNGKQYIISE ELISEGKWVK LEKTTYMDPT GKTRTWESVK

51	RTTRKEQTAD GVAVIPVLQR TLHYECIVLV KQFRPPMGGY CIEFPAGLID

101	DGETPEAAAL RQLEEETGYK GDIAECSPAV CMDPGLSNCT IHIVTVTING

151	DDAENARPKP KPGDGEFVEV ISLPKNDLLQ RLDALVAEEH LTVDARVYSY

201	ALALKHANAK PFEVPFLKF
